# Supplementary material for: Genome-wide Analysis Reveals Extensive Functional Interaction between DNA Replication Initiation and Transcription in the Genome of Trypanosoma brucei
Source: Cell Rep. 2012 Jul 26;2(1):185–97. doi: 10.1016/j.celrep.2012.06.007 (PMC3607257; doi:10.1016/j.celrep.2012.06.007)
Supplement: Document S1. Article plus Supplemental Information [file mmc2.pdf]

Cell Reports
Resource

Cell PRESS
Open ACCESS

Genome-wide Analysis Reveals Extensive Functional Interaction between DNA Replication Initiation and Transcription in the Genome of *Trypanosoma brucei*

Calvin Tiengwe,[1,5] Lucio Marcello,[1,5] Helen Farr,[3,5] Nicholas Dickens,[1] Steven Kelly,[4] Michal Swiderski,[1] Diane Vaughan,[2] Keith Gull,[3] J. David Barry,[1] Stephen D. Bell,[3,*] and Richard McCulloch[1,*]
[1]The Wellcome Trust Centre for Molecular Parasitology
[2]Cellular Analysis Facility
College of Medical, Veterinary and Life Sciences, Institute of Infection, Immunity and Inflammation, University of Glasgow, Sir Graeme Davies Building, 120 University Place, Glasgow G12 8TA, UK
[3]Sir William Dunn School of Pathology, University of Oxford, South Parks Road, Oxford OX1 3RE, UK
[4]Department of Plant Sciences, University of Oxford, South Parks Road, Oxford OX1 3RB, UK
[5]These authors contributed equally to this work
*Correspondence: stephen.bell@path.ox.ac.uk (S.D.B.), richard.mcculloch@glasgow.ac.uk (R.M.)
http://dx.doi.org/10.1016/j.celrep.2012.06.007

SUMMARY

Identification of replication initiation sites, termed origins, is a crucial step in understanding genome transmission in any organism. Transcription of the *Trypanosoma brucei* genome is highly unusual, with each chromosome comprising a few discrete transcription units. To understand how DNA replication occurs in the context of such organization, we have performed genome-wide mapping of the binding sites of the replication initiator ORC1/CDC6 and have identified replication origins, revealing that both localize to the boundaries of the transcription units. A remarkably small number of active origins is seen, whose spacing is greater than in any other eukaryote. We show that replication and transcription in *T. brucei* have a profound functional overlap, as reducing ORC1/CDC6 levels leads to genome-wide increases in mRNA levels arising from the boundaries of the transcription units. In addition, ORC1/CDC6 loss causes derepression of silent *Variant Surface Glycoprotein* genes, which are critical for host immune evasion.

INTRODUCTION

Faithful copying of the genetic material is central to life. However, the features that define origins, the loci at which DNA replication initiation occurs, are poorly understood for most eukaryotes (Masai et al., 2010; Méchali, 2010). Unlike in bacteria, where origins are DNA sequences that show both sequence and positional conservation in different species' genomes (Robinson and Bell, 2005), only in *Saccharomyces cerevisiae* are similarly conserved sequences, termed the autonomously replicating sequence (ARS), found that define the sites of replication initiation (Wyrick et al., 2001). In other eukaryotes consensus sequences at mapped origins are lacking, and in particular in metazoa, it appears that epigenetic cues help define origins, which display an association with transcription the molecular basis of which is as yet unclear. Nevertheless, origins display mechanistic conservation. Eukaryotic chromosomes possess multiple origins, each bound by the six-subunit origin recognition complex (ORC; composed of Orcs 1–6), which recruits the replicative helicase (the MCM complex) via interactions with Cdc6 and Cdt1 (Duncker et al., 2009; Bochman and Schwacha, 2009). Initiation of DNA replication is regulated to occur once per cell division cycle through the actions of cyclin-dependent kinases (CDKs) and the Cdc7/DBF4 kinase complex (Diffley, 2010).

Nuclear DNA replication in African trypanosomes, such as *Trypanosoma brucei*, has been little characterized, and in common with all protists, origins of DNA replication have not been mapped genome wide. Intriguingly, trypanosomes appear to possess only a subset of the higher eukaryotic replication machinery, potentially having a single protein with homology to both Orc1 and Cdc6 (ORC1/CDC6) (Godoy et al., 2009; Tiengwe et al., 2012) and lacking detectable orthologs of Cdc7 and Dbf4 (data not shown). Trypanosomes also have an unusual genome organization, which may provide a particular perspective on replication. In *T. brucei* the majority of protein-coding genes are in 11 diploid megabase-sized chromosomes and are arranged in ~150 "directional gene clusters" (DGCs) that contain, on average, ~50 genes, but can encompass hundreds (Daniels et al., 2010). Transcription of each DGC is thought to initiate from a single RNA polymerase (pol) II promoter, yielding a primary multigene transcript from which individual mRNAs are generated by *trans*-splicing and polyadenylation. Transcription initiates bidirectionally between divergent DGCs (at so-called divergent strand switch regions; SSRs) and at some sites within some DGCs (Martínez-Calvillo et al., 2003; Kolev et al., 2010). These putative promoters display increased abundance of K10-acetylated histone H4 (H4K10Ac), K4-methylated H3, and variants of H2A and H2B (Respuela et al., 2008; Siegel et al., 2009; Wright et al., 2010; Thomas et al., 2009). Termination of transcription is also poorly understood (Martínez-Calvillo et al., 2004), but

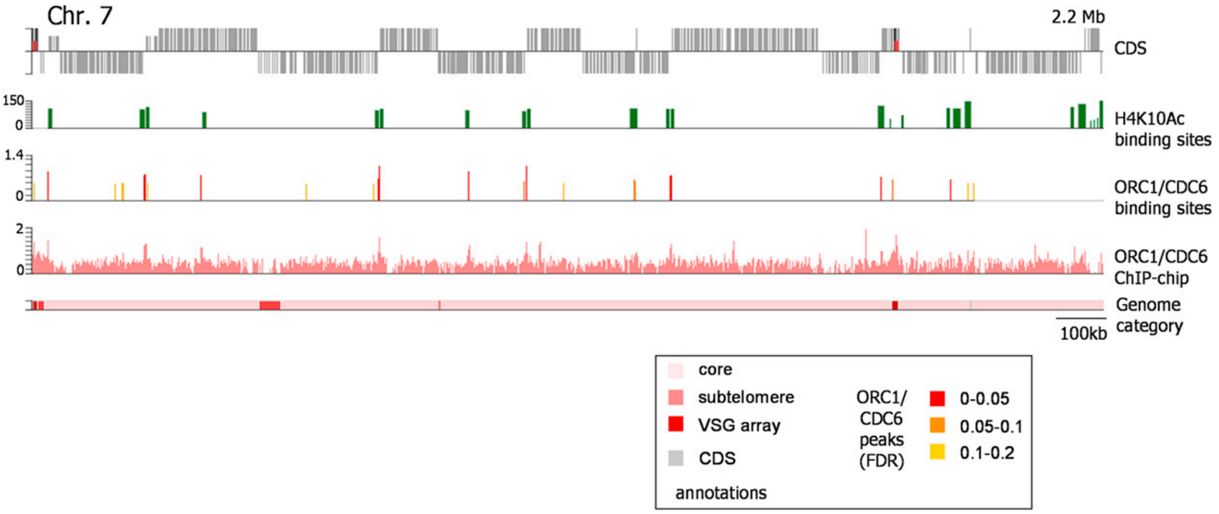

Figure 1. Mapping ORC1/CDC6 Binding in the *T. brucei* Nuclear Genome

To map *T. brucei* ORC1/CDC6 localization sites, ChIP was performed and the recovered DNA cohybridized with input DNA to a microarray; data are shown for chromosome (Chr.) 7. The bottom panel delineates the chromosome into "core," "subtelomere-proximal," and "*VSG* array" sequences (see text for details, and inset for coloring). The location of CDSs (gray boxes) along the chromosome is shown in the top panel; genes above the line are transcribed toward the right, and those below are transcribed toward the left. Enrichment of ORC1/CDC6-bound DNA relative to the input is shown in the second bottom panel; values are plotted as the log2 ratio (y axis) of sample/input and were calculated over a 500 bp sliding window. In the panel above, predicted ORC1/CDC6 binding sites are identified as "peaks," which are shown by vertical lines colored to indicate the likelihood of being an ORC1/CDC6 binding site based on three categories of FDR: red indicates the highest confidence (FDR $\leq$0.05), and orange and yellow decreasing confidence (FDRs of 0.05–0.1 and 0.1–0.2, respectively). In the second top panel, sites of H4K10Ac localization are indicated as green vertical lines; these data are derived from ChIP-seq data of Siegel et al. (2009), identifying positions of likely transcription start sites, and are represented as log2 values (y axis) in 250 bp windows, with the width of the lines indicating the areas of the chromosome covered by the modified histone. See also Figure S1.

the ends of DGCs (which are frequently, but not exclusively, convergent SSRs) display increased abundance of H3 and H4 variants (Siegel et al., 2009). A hypermodified base, J, also localizes to the boundaries of the DGCs (as well as telomeres, other repeats, and inactive *Variant Surface Glycoprotein* [*VSG*] expression units; see below) and influences transcription (Cliffe et al., 2010; Ekanayake et al., 2011).

The *T. brucei* nuclear genome also has hundreds of *VSG* genes, switching between which is the basis of antigenic variation, a strategy for evasion of mammalian immunity. Transcriptionally silent *VSG*s are located proximal to telomeres in ~100 nuclear minichromosomes (Wickstead et al., 2004) and, more abundantly, in arrays in the subtelomeres of the megabase chromosomes (Marcello and Barry, 2007). To be expressed in the mammal, *VSG*s are recombined into 5–15 bloodstream *VSG* expression sites (BESs) at the telomeres of the megabase and intermediate chromosomes; only one BES is normally actively transcribed at a time (Horn and McCulloch, 2010). BES transcription is multigenic, with the *VSG* cotranscribed with approximately ten expression site-associated genes (*ESAG*s) (Hertz-Fowler et al., 2008). Another set of *VSG*s, termed the *MVSG*s, is expressed in the nonreplicating metacyclic trypanosome form, a transmission stage found in the tsetse fly vector. *MVSG*s are transcribed from ESs (MES) that lack *ESAG*s and are thus the only single-gene *T. brucei* transcription units (Ginger et al., 2002). All *VSG* transcription is mediated by RNA pol I.

Here, we present a global analysis of DNA replication initiation in *T. brucei* and show that DNA replication and transcription display remarkable levels of coordination, both positionally and functionally. We map ORC1/CDC6 binding sites and show that they localize to the boundaries of the transcribed DGCs in the chromosome cores. In contrast, we see high-density binding in the silent *VSG* array-containing subtelomeres. Mapping DNA replication initiation shows that early origins colocalize with a subset of the ORC1/CDC6 sites in the chromosome cores and display a spacing that is greater than in other eukaryotes. Finally, using RNA interference of ORC1/CDC6, we show that DNA replication functionally intersects with transcription because the absence of the initiator leads to increased levels of transcripts from the DGC boundaries and derepression of *MVSG* silencing.

RESULTS

Genomic Distribution of the Replication Initiator ORC1/CDC6

To address how DNA replication occurs in the unusual *T. brucei* genome, we first profiled the genomic binding sites for the candidate initiator protein ORC1/CDC6 in procyclic form (PCF; tsetse midgut stage) cells. A hemizygous *ORC1/CDC6 T. brucei* strain was generated in which one allele was deleted and the other modified to encode a C-terminal 12 Myc epitope-tagged ORC1/CDC6 variant (data not shown). Chromatin immunoprecipitation (ChIP)-chip was then performed using the myc tag, interrogating an ~385 K microarray covering the *T. brucei* TREU927 megabase chromosomes. Figures 1 and 2 show

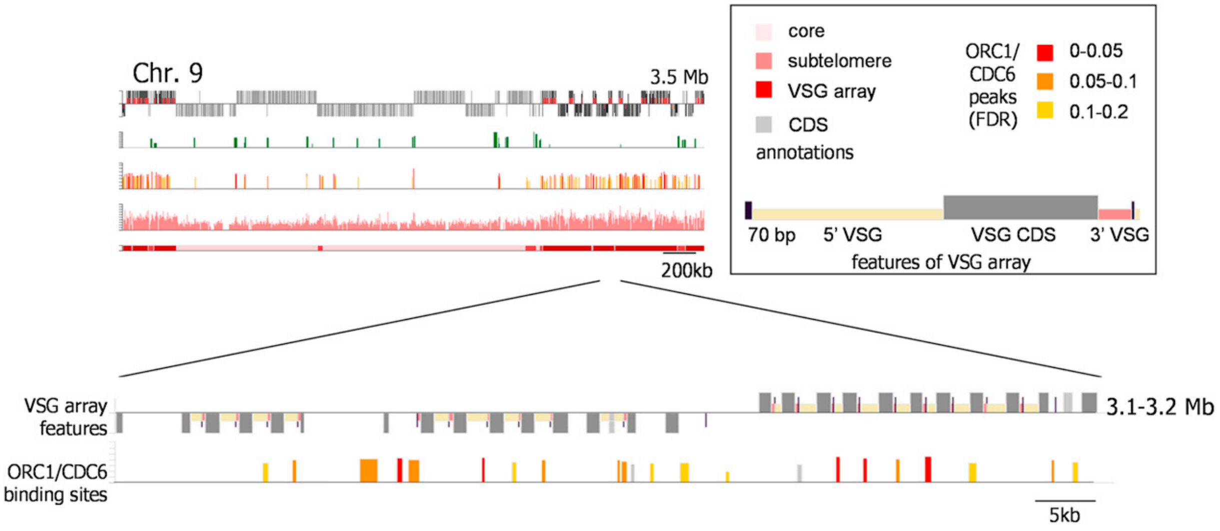

Figure 2. Mapping ORC1/CDC6 Binding in the *T. brucei* VSG Arrays

ORC1/CDC6 localization is shown for chromosome (Chr.) 9, as detailed in Figure 1, focusing on an ~100 Kb portion of the subtelomeric *VSG* array (the location of this region within the whole chromosome is indicated). Annotation of specific sequence elements of the *VSG* "cassettes" is identified in the inset: the *VSG* CDSs are gray boxes, 70 bp repeats are black boxes, and 5′ and 3′ *VSG* sequences are smaller yellow and pink boxes, respectively. Predicted ORC1/CDC6 binding sites (at three levels of FDR) are shown relative to these *VSG* elements. See also Figure S1.

ORC1/CDC6-enriched sites in chromosomes 7 and 9, which are representative of all 11 chromosomes (Figure S1). As for ORC in other organisms (Eaton et al., 2010; MacAlpine et al., 2010), *T. brucei* ORC1/CDC6 ChIP enrichment (up to ~4-fold) is low relative to that of other DNA binding proteins, such as modified histones (H4K10Ac is shown in Figures 1 and 2) or transcription factors (Siegel et al., 2010; Thomas et al., 2009). Applying a false discovery rate (FDR) of ≤0.2, 953 ORC1/CDC6 binding sites were predicted, covering ~640 Kbp (2.4%) of the megabase chromosomes. This revealed a striking, bimodal binding pattern, with dispersed ORC1/CDC6 binding in the interior of chromosomes (Figure 1) and denser binding at subtelomeres (Figure 2).

To analyze ORC1/CDC6 distribution globally, each megabase chromosome was demarcated into three categories (Figure 3A): "chromosome core," the highly transcribed region containing housekeeping genes in DGCs as found in all chromosomes; "subtelomere-related," a combination of nonfunctional and functional elements (e.g., *ESAG* [pseudo]genes, *VSG*-related genes, and *RHS* genes); and "*VSG* array," which is also subtelomeric but has a more ordered arrangement, containing arrayed "cassettes" (Figure 2) composed of 70 bp repeats, *VSG 5′*-flanking sequence, *VSG* ORF (either functional or pseudogene), and *VSG 3′* flank. The core (total size 22.3 Mbp, 73% probe coverage) housed 38% (362) of ORC1/CDC6 binding sites (Figure 3A), with an average spacing of ~130 Kbp between adjacent members of 170 "unique" binding sites (those closer than 10 Kbp were grouped). In contrast 51% (484) of binding sites were in the *VSG* arrays (2.1 Mbp, 61% probe coverage), at an average spacing of ~7 Kbp. Subtelomere-related sequence (1.3 Mbp) is underrepresented by probes (29% coverage), and therefore, the 11% of binding sites (107) at ~12 Kbp spacing may be an underestimate.

Within the chromosome cores the ORC1/CDC6 binding sites clustered at the boundaries of the *T. brucei* multigenic transcription units. The promoters of these units have been inferred by H4K10Ac localization (Siegel et al., 2009), which identifies both divergent SSRs and intra-DGC transcription start sites. Thirty-eight percent of core ORC1/CDC6 binding sites (136 in total, mapped at FDR ≤0.2) mapped to divergent SSRs (Figure 3B), of which 62 (70%) out of the predicted 88 such loci showed evidence of at least 1 ORC1/CDC6 binding site. Fifty-four percent of ORC1/CDC6 sites (202 total, FDR ≤0.2) fell within DGCs, and virtually all localized to predicted transcription start sites: of the predicted 46 intra-DGC loci marked by H4K10Ac localization, 44 (96%) showed evidence of ORC1/CDC binding. Taken together, these data suggest an exceptionally strong association between ORC1/CDC binding and transcription initiation. Despite this close correlation in genome-wide localization (Figures 1, 4, and S1), a functional relationship between H4K10Ac modification and ORC1/CDC6 recruitment seems unlikely. We examined the localization of H4K10Ac by merging those of the predicted 367 binding sites that were closer than 10 Kbp, and discarding those that were <1 Kbp in size, yielding 158 unique binding loci. Of these, 108 were found within 15 Kbp of a predicted ORC1/CDC6 site, and 92 overlapped with at least 1 ORC1/CDC6 site. However, the two binding "footprints" do not overlap entirely. At 70% of ORC1/CDC6 sites within a DGC and proximal (within 10 Kbp) to H4K10Ac, the initiator binding site preceded the modified histone (examples can be seen at 0.35 and 1.25 Mbp in chromosome 7; Figure 1). In addition within divergent SSRs, where H4K10Ac localization is frequently seen as two distinct sites (e.g., 0.25, 0.71, and 1.0 Mbp in chromosome 7; Figure 1) (Siegel et al., 2009), ORC1/CDC6 binding was found centrally, between these sites. Thus, ORC1/CDC6 binding appears to localize close to H4K10Ac, but somewhat

Cell PRESS
Open ACCESS

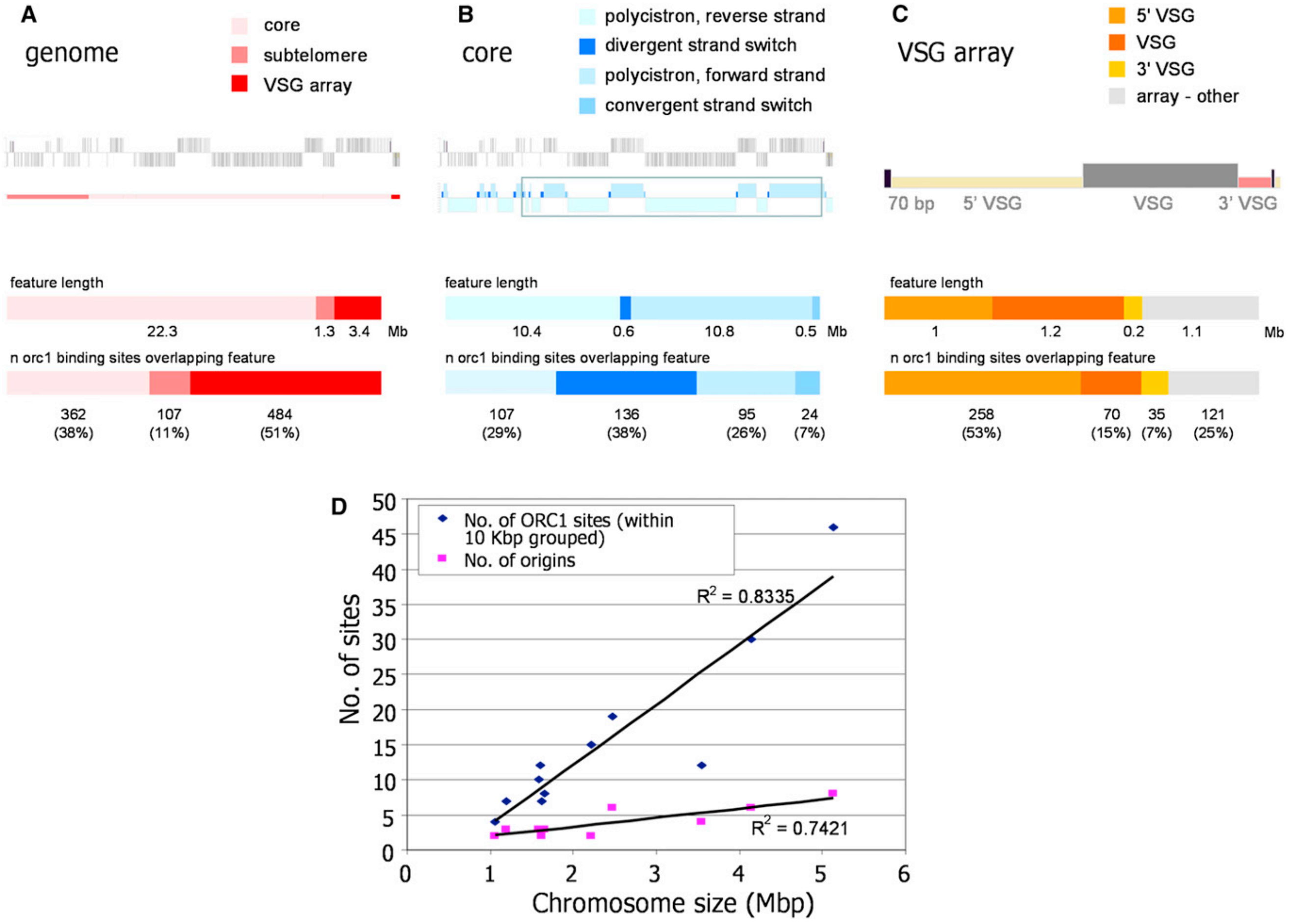

Figure 3. Localization of ORC1/CDC6 in the *T. brucei* Nuclear Genome

(A) Each chromosome (2 is shown as an example; see Figure 1 for explanation) was delineated into core (light pink), subtelomere-proximal (pink), or *VSG* array (red). The amount of sequence each category represents genome wide is graphed, as is the number (n) of ORC1/CDC6 (ORC1)-predicted binding sites in each category.

(B) The chromosome cores were next delineated into DGCs (polycistrons) in which the CDS are transcribed toward the right (forward strand; pale blue) or left (reverse strand; very pale blue), or into divergent (dark blue) or convergent (midblue) SSRs. Graphs show the amount of sequence encompassed by each of these regions, and the number of predicted ORC1/CDC binding sites that fall within them.

(C) For the *VSG* arrays, specific sequence components of the *VSG* cassette were delineated (see Figure 2). The amount of sequence in the *VSG* arrays that correspond with the *VSG* ORF (functional or pseudogene; dark orange), the *VSG* 5′ flank (light orange), the *VSG* 3′ flank (yellow), or not recognizably *VSG* cassette related (other; light gray), is shown in the upper graph, whereas the lower graph shows the number of predicted ORC1/CDC binding sites that fall within each category (note that 70 bp repeat sequence was not mapped because it is masked in the microarray approach adopted).

(D) The number of predicted ORC1/CDC6 binding sites (those within 10 Kbp are grouped as a single site) and the number of predicted early replication origins (see Figure 4) are shown relative to the size of each megabase chromosome.

See also Figure S2.

upstream. It is also clear that the transcription initiation is not the sole determinant of ORC1/CDC6 binding: though only 7% of ORC1/CDC6 binding sites mapped to convergent SSRs, 24 of 64 such loci show evidence for ORC1/CDC6 binding (FDR $\leq$0.2; 40%), and some clearly act as early replication origins (see below). It therefore seems likely that ORC1/CDC6 binds to all transcription boundaries, but less efficient localization to terminators is not always detected by ChIP-chip.

To investigate the high density of ORC1/CDC6 binding in the *VSG* arrays, individual sequence components (*VSG* 5′ flank, *VSG* ORF, and *VSG* 3′ flank), comprising 69% of the arrays, were demarcated, and the extent of binding was compared (Figures 2 and 3C; FDR $\leq$0.2). *VSG* 5′ flank and *VSG* ORF comprise the majority (40% and 50%, respectively) of *VSG* array sequence. ORC1/CDC6 sites were overrepresented in the 5′ flank (53% of sites) and underrepresented in the *VSG* ORF (15%), whereas *VSG* 3′ flank sequences bound the protein at a frequency commensurate with their contribution to the overall arrays. Despite these nonrandom distributions, we could not identify a consensus binding sequence (Figure S2), suggesting that ORC1/CDC6 binding in the *VSG* arrays, like in the core, is not a sequence-specific feature. Indeed, because the *VSG*

Cell PRESS Open ACCESS

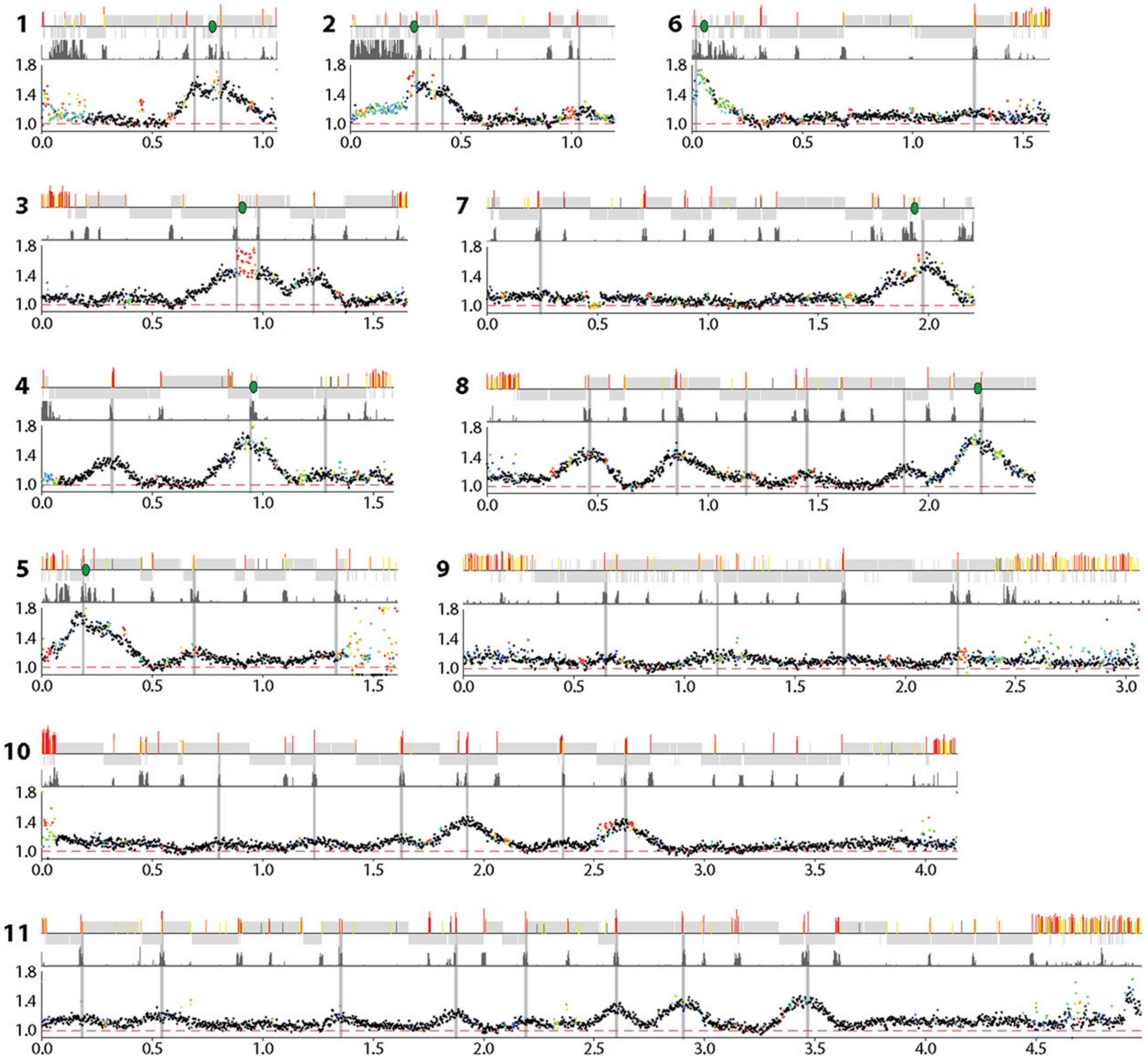

Figure 4. Mapping Replication Origins in the *T. brucei* Nuclear Genome

Graphs show the distribution of replication origins in the megabase-sized chromosomes (numbered 1–11; numbers on the x-axis denote sizes in Mbp), determined by the extent of enrichment of DNA in S phase relative to G2. For each chromosome the top track displays CDSs (gray bars), and the relative position of predicted ORC1/CDC6 binding sites (color coding described in Figure 1) and centromeres (green ovals for chromosomes 1–8). The second track displays localization of H4K10Ac, derived from ChIP-seq data of Siegel et al. (2009), identifying positions of likely transcription start sites. The graphs in the lowest track display the ratio of the read depth between the S phase and G2 samples, where each dot represents 2,500 bp. The color of the dot refers to the mapping quality score at that position: black shows a score ≥50, whereas a heatmap of blue to red shows a score of 49–0. See also Figure S3.

arrays are not normally transcribed and lack known promoters and terminators, it is conceivable that ORC1/CDC6 localization in this region of the genome is functionally distinct from that in the core.

Identification of Early Replication Origins in the *T. brucei* Genome

To address whether or not the mapped ORC1/CDC6 binding sites correspond with replication origins, we employed marker frequency analysis (MFA). This technique compares the copy number of marker sequences in replicating and nonreplicating cells; origin proximal sequences will be overrepresented compared with origin-distal markers during replication. Early-mid S phase and G2-enriched populations of PCF *T. brucei* cells were prepared by cell sorting (data not shown). Following library preparation and Illumina sequencing, sequence reads were mapped to the *T. brucei* TREU927 genome; read abundance was then calculated in 2,500 bp windows as the ratio of replicating to nonreplicating DNA, revealing peaks of varying amplitude (Figure 4). We apply the following nomenclature to these

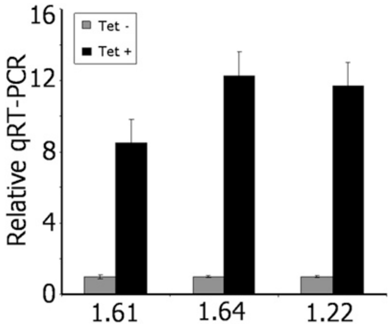

Figure 5. RNAi of ORC1/CDC6 Leads to Metacyclic *VSG* mRNA Expression in PCF *T. brucei*

Expression levels of three *MVSG* genes (1.61, 1.64, 1.22) were measured in PCF *T. brucei* cells by qRT-PCR, comparing mRNA levels for each relative to *GPI8* (as a control) before (Tet −, gray bar) and after induction (Tet +, black bar) of RNAi against ORC1/CDC6. In each case the level of mRNA of the uninduced sample is shown as one and the mRNA level in the RNA-induced sample shown relative to that; vertical lines indicate SD from three experiments. See also Figure S4.

peaks; OBR (Origin of Bidirectional Replication): Chromosome Number: Peak position (in megabase pairs from the left end of the chromosome). Thus, the second peak from the left end of chromosome 11 (at 0.55 Mbp) will be referred to as OBR:11:0.55. In parallel with the MFA sequencing (MFAseq), we performed MFA using qPCR on independently sorted cells, using a range of primer pairs to cover a selection of chromosomes. These data concur with the MFAseq data (Figure S3). In total, 44.7% of the genome showed a degree of replication in these assays, in good agreement with observations in other eukaryotes where a temporal hierarchy of origin firing exists (Masai et al., 2010). The cell sorting performed to obtain the S phase population will have enriched for origins that fired in the first 50% of S phase. Thus, late-firing origins will be underrepresented or absent from our analysis. Nevertheless, several key observations can be made.

First, and most strikingly, the peaks observed are broad and well spaced, indicating a low density of active origins. We observed a total of 42 peaks of varying heights and found a correlation between number of origins and chromosome length (Figure 3D). Extrapolating to the whole megabase chromosome genome, we predict a total of ∼100 origins, giving an origin density of 1 origin per ∼260 Kbp, significantly sparser than budding yeast (1 origin per 46 Kbp) (Yabuki et al., 2002), *Arabidopsis* (1 origin per 77 Kbp) (Costas et al., 2011), or mammals (1 origin per 25 Kbp–1 origin per 130 Kbp) (Sequeira-Mendes et al., 2009; Cayrou et al., 2011). Second, all origins lie within the chromosome cores. Thus, as in other eukaryotes, telomere-proximal regions are late replicating: 95.5% of the replicated regions fell within the chromosome cores, with only 4% in subtelomeric regions and 0.5% in the *VSG* arrays (Figure S3). Third, some peaks are symmetrical, whereas others are not. Symmetry indicates bidirectional replication at the same rate in both directions, and these peaks were found at divergent and convergent SSRs (e.g., OBR:8:1.45 and OBR:8:1.9, respectively). Asymmetric peaks, in contrast, were found at intra-DGC sites where transcription both initiates and terminates. In all cases the slope of the peak was shallower when the direction of replication follows transcription and steeper when replication direction is counter to that of transcription. Taking the example at OBR:8:0.86 (Figure 4), replication proceeds 2.1-fold faster when traveling in the direction of transcription than counter to it. This indicates that *T. brucei* does not avoid head-on encounters between transcription and replication (Figure S3) and that such encounters impose a greater impediment to replication than when the two processes are codirectional (Merrikh et al., 2011). Fourth, there is a perfect correlation between the position of the mapped centromeres of *T. brucei* chromosomes (to date, described in chromosomes 1–8) Obado et al., 2007) and the MFA peaks of highest amplitude. Thus, *T. brucei* replicates centromere-proximal loci early in S phase. Fifth, 39 of the 42 MFA peaks center on ORC1/CDC6 binding sites, supporting the proposed function of ORC1/CDC6 as an initiator protein. Nonetheless, it is clear from the two data sets that a large number of ORC1/CDC6 binding sites (∼80% of the 170 sites grouped with 10 Kbp) do not correlate with active origins of replication (Figure 3). Although some of these sites may account for late-firing origins, there are many instances where binding sites lie within regions replicated from adjacent OBRs, suggesting potential redundancy.

Detailed examination of the positions of the OBRs reveals a striking relationship between the localization of origins and the transcribed domains. Most obviously, 19 of the peaks centered on divergent SSRs, correlating with sites of promoter activity. An additional three peaks were found at convergent SSRs, which were not always scored as significant ORC1/CDC6 binding sites (e.g., OBR:8:1.89) (Figure 4), consistent with our suggestion that the initiator binds transcription termination sites. Of the remaining 20 peaks, 2 were found in regions with a more broken distribution of genes between the 2 strands and 18 within DGCs, all at regions of H4K10Ac enrichment, indicative of transcription initiation and termination sites. Thus, all the observed origins coassociate with the boundaries of transcription.

ORC1/CDC6 Influences Transcription

In light of the association of *T. brucei* ORC1/CDC6 binding sites and origins with the boundaries of transcription units, and also the extremely high density at subtelomeric regions, we wished to address a possible role in transcriptional processes. We therefore examined mRNA abundance after ORC1/CDC6 RNAi. We first looked directly for a role in *VSG* expression by quantitative RT-PCR (qRT-PCR) (Figure 5). In *T. brucei* strain EATRO795 (Ginger et al., 2002), four *MVSG*s and promoters have been detailed and shown to be transcriptionally repressed in PCF cells. We therefore modified these cells to allow tetracycline-controlled induction of RNAi and introduced an *ORC1/CDC6* RNAi construct. As described previously by Godoy et al. (2009), inducing RNAi against ORC1/CDC6 led to only modest growth inhibition after 96 hr, despite loss of ∼70% of ORC1/CDC mRNA (data not shown). At the same time point post-RNAi induction, significant increases (5- to 13-fold) in *MVSG* mRNA levels were seen (Figure 5), suggesting that loss of ORC1/CDC6 leads to derepression of the MESs. To ask if this

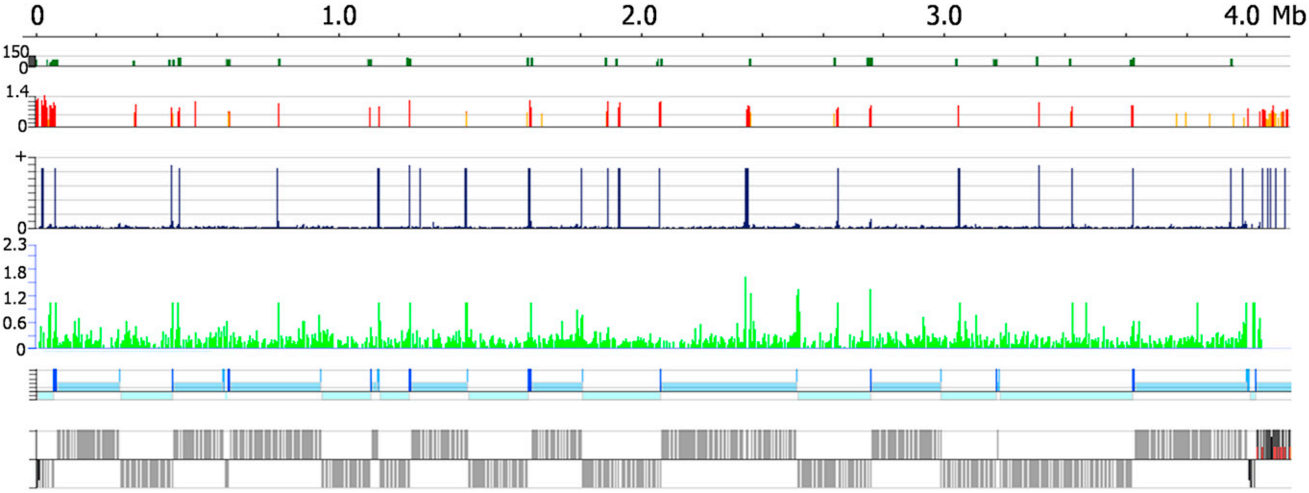

Figure 6. ORC1/CDC6 RNAi Leads to Changes in mRNA Abundance throughout the Genome in *T. brucei*
The effect of ORC1/CDC6 RNAi on mRNA abundance is shown for all predicted genes in chromosome 10. Top panel shows H4K10Ac binding sites as peaks (green lines); the y axis shows max log2 ratio in 250 bp windows. Second panel illustrates predicted locations of ORC1/CDC6 binding sites (red, orange, or yellow lines, as in Figure 1). Changes in mRNA abundance following ORC1/CDC6 RNAi are compared by two methods: the third panel shows regions where RNAi results in detectable RNA sequence tags in the RNAi-induced sample but not in the uninduced (vertical blue lines; height is arbitrary, and width is shown in 250 bp windows); the fourth panel shows the change in RNA sequence tag abundance in the RNAi-induced sample relative to the uninduced sample across the chromosome (green vertical lines denote fold change of induced/uninduced as log2 values [y axis], mapped in 250 bp windows). Fifth panel details the direction of transcription of the genes (light blue indicates a DGC transcribed toward the right, and very light blue a cluster transcribed toward the left) and highlights the positions of convergent (midblue box) and divergent (dark-blue box) SSRs. Bottom panel presents the locations of CDS (gray boxes). See also Figure S5 and Table S1.

was true also for the BESs and in bloodstream form (BSF; mammal-infective) parasites, we analyzed RNAi in *T. brucei* strain Lister 427, where 14 distinct *BVSG*s in 15 BESs have been cataloged by Hertz-Fowler et al. (2008). In PCF cells of this strain, ORC1/CDC6 RNAi (70% mRNA loss) led to the same slow growth as in EATRO795 PCF cells (data not shown). However, derepression of the normally silent BESs was much less marked than seen for MESs (Figure S4A): of five *BVSG* genes examined, only three displayed significantly increased mRNA abundance, and only by 1.5- to 2.5- fold. In the BSF Lister 427 (90-13) cells used here, a single BES (427-2, expressing *VSG221*) is maximally transcribed, and the others are silent. In this life cycle stage, *ORC1/CDC6* RNAi has a more profound effect on *T. brucei* survival, with cell death after 36–48 hr (Tiengwe et al., 2012). For this reason, *BVSG* mRNA abundance was measured 12 hr post-RNAi induction, at which point ~40% loss of ORC1/CDC6 mRNA was seen. No evidence was found for derepression of the silent BESs (Figure S4B).

To address whether the aforementioned response is due to locus-specific functions of ORC1/CDC6 or reflects a genome-wide influence on gene expression, we used RNA sequencing (RNAseq) to compare mRNA abundance in PCF Lister 427 cells 96 hr post-RNAi induction relative to uninduced cells. Read abundance was calculated in 250 bp windows across the genome and the extent of increased mRNA in the ORC1/CDC6 RNAi-induced sample relative to the uninduced sample examined as a function of position in the TREU927 genome. In many regions, transcripts were seen from loci where there was no detectable gene expression before ORC1/CDC6 RNAi; this is shown in Figure 6 for chromosome 10, which is representative of the whole genome (Figure S5). We also tested for loci that showed changes in mRNA abundance, from a baseline of detectable mRNA before RNAi, with strikingly similar findings (chromosome 10 is shown in Figure 6). These analyses revealed that ORC1/CDC6 RNAi had a marked, nonrandom effect on global gene expression, with increased mRNA abundance for genes positioned proximal to the boundaries of the transcription units. These changes matched closely the localization of ORC1/CDC6 protein, with increased mRNA from the vicinity of divergent SSRs (e.g., ~0.42 and 1.22 Mbp on chromosome 10), convergent SSRs (e.g., ~1.40 and 1.80 Mbp), and intra-DGC initiation/termination sites (e.g., ~0.80 and 2.35 Mbp) (Figure 6). The increased mRNA abundance was not greater at the regions of early origin firing (e.g., four origins are found in chromosome 10; OBR:10:0.80, OBR:10:1.25, OBR:10:1.92, and OBR:10:2.65; Figure 4), suggesting that it is not linked to replication.

The changes in transcript abundance did not normally result from elevated expression of genes within the DGCs (and proximal to the ends) but resulted mainly from increased abundance of RNA sequence reads upstream and downstream of the transcription start and stop points. However, in some cases, ORC1/CDC6 depletion did appear to undermine functionally significant gene expression controls (Table S1; genes showing significant changes in mRNA abundance; >1.4-fold), with increased transcript abundance of genes positioned at the end of both RNA pol I (e.g., *PAG* and *GRESAG2*) and pol II transcription units (e.g., *ISG65*, numerous *PAG*-like genes, and a putative iron/ascorbate reductase-encoding gene cluster). Expression of the *ISG* and *PAG* genes is known to be under transcriptional

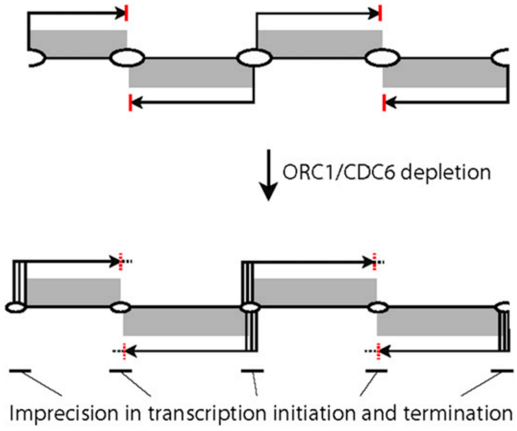

Figure 7. Functional Interaction between DNA Replication and Transcription in *T. brucei*

A model of the effect of ORC1/CDC6 depletion on transcription and replication is shown. DGCs are shown as gray boxes, with origins of bidirectional replication as ovals. Transcription start sites are shown as vertical black lines, transcripts and their direction as horizontal black arrows, and termination sites as vertical red bars. Upon ORC1/CDC6 depletion, replication initiation efficiency reduces (small ovals), and termination barriers become more porous (red dotted lines), allowing a proportion of transcripts to elongate through them (dotted horizontal lines). In addition, start site definition becomes less precise (clustered vertical black lines). The consequence of this lack of precision in both transcription start and termination sites is elevated levels of transcripts at the boundaries of the DGCs.

control (Haenni et al., 2009; Leung et al., 2011), which at least for *PAG* is based on locus-specific termination or elongation control. Loss of ORC1/CDC6 may therefore result in readthrough transcription in all these cases. Increased RNA sequence read abundance after ORC1/CDC6 RNAi was also observed from the *VSG* arrays (Figures 6 and S5), which emanated from intergenic sequences, and not from the *VSG* CDS (data not shown). However, sequence differences between the *VSG*s of *T. brucei* strain Lister 427 (which it was necessary to use for RNAi) and TREU927 (where the *VSG* array genes have been positionally annotated) undermine attempts to map *VSG* RNAseq reads, and so we cannot discount the possibility that ORC1/CDC6 RNAi has global effects on *VSG* expression.

DISCUSSION

The data in this study reveal remarkably precise localization of the replication initiator ORC1/CDC6 to the boundaries of transcribed domains in the core of the diploid megabase chromosomes that constitute much of the *T. brucei* nuclear genome. In addition the data show that these ORC1/CDC6 binding sites act as replication origins, albeit with considerable redundancy, and that the coordination of transcription and replication is not simply positional. Following ORC1/CDC6 RNAi we show that transcript abundance is elevated upstream and downstream of the transcription units and occasionally affects gene expression within these units. ORC1/CDC6 therefore contributes to the global delineation of the transcription boundaries in *T. brucei* (Figure 7). Thus, analyzing this highly unusual genome has revealed an unprecedented level of interplay between transcription and DNA replication in a eukaryotic genome. It appears that both these crucial cellular reactions are delineated into discrete domains, most likely through the functional interaction and co-localization of the underlying machineries. We propose that this functional organization of the genome into modules is possible as a consequence of the near-exclusively posttranscriptional basis of gene regulation in kinetoplastid organisms and may have far-reaching consequences for genome maintenance. Moreover, our identification of the coassociation of highly active and early-firing replication origins with centromeres may contribute to the development of stably inherited extrachromosomal DNA molecules in *T. brucei*, thereby facilitating many forms of experimental analysis.

In fission and budding yeast, as well as in *Drosophila melanogaster* and *Arabidopsis thaliana*, origins have been identified through genome-wide mapping of the binding of ORC and MCM subunits by ChIP and by mapping sites where DNA synthesis begins by trapping newly formed replication forks or by isolating nascent leading strands (Gilbert, 2010). In mammals, ChIP of pre-RC components has not been reported, and therefore, origin identification has relied on initiation site mapping. Our work combines ORC1/CDC6 ChIP with MFA to define origins in *T. brucei*, adding information gleaned from this highly diverged protist to our knowledge of what constitutes an origin in eukaryotes, revealing both similarities and differences. In common with most other eukaryotes, we have not been able to identity consensus binding sequences for *T. brucei* ORC1/CDC6 (data not shown), perhaps suggesting that epigenetic features underlie ORC1/CDC6 recruitment to origins (see below). *Schizosaccharomyces pombe* origins are found in AT-rich intergenic sequences (Dai et al., 2005; Hayashi et al., 2007), due to ORC DNA binding via an AT-hook domain on Orc4 (Chuang and Kelly, 1999). A preference for AT-rich sequences has also been reported for *D. melanogaster* ORC (MacAlpine et al., 2004), which additionally appears to localize predominantly at active promoters with open chromatin structure (MacAlpine et al., 2010), a feature of origins also observed in *S. cerevisiae* (Eaton et al., 2010). This appears compatible with *T. brucei* ORC1/CDC6, which shows preferential binding to genome regions that house the poorly defined promoters at the head of the multigene transcription units. These loci are marked by the deposition of variant and modified histones and display more mobile nucleosomes (Siegel et al., 2009). However, the association between origins and promoters is not absolute in eukaryotes. For instance in *D. melanogaster* a further study, mapping nascent strands, did not find enrichment at transcription start sites but instead at CpG-rich sequences frequently found in gene exons (Cayrou et al., 2011). CpG sequences may also be a feature of mammalian origins, which appear to be enriched at promoters, though are also found frequently within genes (Cadoret et al., 2008; Karnani et al., 2010; Cayrou et al., 2011; Sequeira-Mendes et al., 2009). Finally, *A. thaliana* ORC and CDC6 binding, and origin activity, have a preference for the 5′ body of genes (Costas et al., 2011). For *T. brucei*, ORC1/CDC6 displays localization to the ends of the transcription units, and these loci can act as origins, though with less efficiency than ORC1/CDC6 sites at the head of the transcription units. To our

knowledge, origin activity at transcription terminator regions has not previously been seen.

Although our data reveal that replication and transcription in *T. brucei* functionally intersect, the basis for this is unclear, as is the mode of ORC1/CDC6 recruitment to origins. In *A. thaliana*, origins are associated with CG-poor sequences that are enriched for variants of histone H3 di- and trimethylated on lysine residue 4 (H3K4), as well as the H2A.Z variant (Costas et al., 2011). *D. melanogaster* ORC binding sites appear to be enriched in histone H3.3, and perhaps H2Av, variants (MacAlpine et al., 2010), whereas mono-methylation of H4K7 in mammals has been implicated in pre-RC assembly at origins (Tardat et al., 2010). In *T. brucei*, trimethylated H3K4 and H2A.Z each localize to transcription start sites (Siegel et al., 2009; Wright et al., 2010), perhaps suggesting some conservation in ORC localization with *A. thaliana*. However, the variant histones that have been mapped to date at transcription initiation and termination sites in *T. brucei* are distinct (Siegel et al., 2009), and our mapping and understanding of the function of all *T. brucei* histone modifications are undoubtedly incomplete (Figueiredo et al., 2009). We also cannot yet exclude other, nonchromatin modes of ORC recruitment to DNA (Donti et al., 2009). Thus, it is premature to say if epigenetic features that are common to the ends of the DGCs define ORC1/CDC6 binding and, indeed, are conserved with other eukaryotes. For the same reason further work will be needed to understand if the depletion of ORC1/CDC6 causes transcription changes indirectly, for instance due to changes in chromatin through loss of interaction, or if the *T. brucei* initiator plays a more direct role in transcriptional events (Sasaki and Gilbert, 2007).

A particularly striking feature of the replication profile of *T. brucei* lies in the relative paucity of replication origins. We estimate that fewer than 100 origins account for replication of the entire complement of megabase chromosomes of *T. brucei*. As discussed above, this is a far lower density of origins than seen in other eukaryotes. It is possible that the number of origins is constrained in part by the architecture of the megabase chromosomes, with their long DGCs. In this context it would seem intuitive that origins would localize to divergent SSRs, thus preventing clashes between transcription and replication machineries. Both bacteria and other eukaryotes have chromosome architectures that limit such conflicts. For example the rDNA loci in *Escherichia coli* are arranged in the same orientation as that of replication fork passage. Similarly, in yeasts and mammals specific replication fork barriers exist in the rDNA loci to prevent clashes between transcription and replication machineries (Labib and Hodgson, 2007). Interestingly, in trypanosomes we observe several examples where replisome and RNA pol are in opposition, resulting in a marked slowing of the rate of replication fork progression (Figure S3). Thus, the low number of origins cannot solely be accounted for by architectural constraints. An alternative explanation lies in the possibility that trypanosomes branched early in the eukaryotic tree of life. Recently, Diffley has proposed that the increase in the number of replication origins required to replicate the increasingly large genomes that arose during eukaryotic evolution has resulted in selection for additional levels of control circuitry (Diffley, 2011). The apparent redundancy of control circuits in more complex eukaryotes reflects a fail-safe situation to ensure once per cell-cycle replication at any given origin. It is notable, therefore, that whereas trypanosomes possess cyclin and Cdk homologs, they may lack both the important Cdc7/Dbf4 and geminin/Cdt1 systems found in more recently branching eukarya. Perhaps the limited control circuitry of trypanosomes has constrained the number of origins that can be employed to replicate their genome.

The small number of replication origins in *T. brucei* raises some further questions. The first of these relates to the excess of ORC1/CDC6 binding sites relative to origins. Even accounting for the fact that we have mapped to date only the origins that we estimate fire in the first half of S phase, it is likely that there are ~2-fold more ORC1/CDC6 sites than the 100 origins. Furthermore, it seems likely that some origins fire earlier, or more readily, than others, based on the variable sizes of the MFA peaks we map. It is well known in other eukaryotes that ORC binding sites are more abundant than origins and that a temporal order of origin firing exists (Masai et al., 2010; Douglas and Diffley, 2012). The basis for this remains unclear, but it is likely that early origins have a higher affinity for replication factors. In the context of the *T. brucei* genome, where it is likely that all promoters are equally active and initiate transcription constitutively, how differences emerge in the efficiency of ORC1/CDC6 binding or downstream replication events is unclear. The second question relates to replication of the *VSG* array-containing subtelomeres. Although we find exceptionally high density of ORC1/CDC6 binding in the *VSG* arrays, we see no evidence for origin activity. It is possible that, as in other eukaryotes such as *Schizosaccharomyces pombe* (Hayashi et al., 2007), these subtelomeric regions are late replicating and have not been captured by the MFAseq. Alternatively, the high-abundance ORC1/CDC6 sites may act as many dormant origins (Blow et al., 2011), perhaps because replication of these chromosome domains is distinct from the cores due to the lack of *VSG* array transcription. A final possibility is that ORC1/CDC6 provides a distinct, nonreplication function in this chromosome domain. Whether it acts as a silencer, as in yeast and mammals (Prasanth et al., 2010; Hickman and Rusche, 2010), is unknown, but this may be compatible with the increased expression on *MVSG* genes after ORC1/CDC6 RNAi that we observe. Indeed, Orc1 in *Plasmodium falciparum* displays localization to telomeres (Mancio-Silva et al., 2008) reminiscent of what we describe here in *T. brucei*. Because both parasites populate their telomeres with genes central to antigenic variation, this perhaps suggests a common connection between immune evasion and the ORC machinery.

EXPERIMENTAL PROCEDURES

T. brucei PCF cells were of strain TREU927, Lister 427, or EATRO795 and were grown at 27°C in SDM-79 medium. BSF cells were of strain Lister 427 and were grown in HMI-9 medium at 37°C. To perform RNAi, *T. brucei* PCF strain 427 pLew29-pLew13, and BSF strain pLew90-pLEW13, developed by Wirtz et al. (1999), were used, constitutively coexpressing T7 RNA pol and Tet repressor. To generate a PCF derivative of EATRO795 in which RNAi could be performed, the individual pLew29 and pLew13 plasmids were introduced by transformation, and controllable expression was tested by monitoring expression of a reporter gene subsequently introduced under the control of

the T7 RNA pol promoter (data not shown). To generate cells for RNAi, a fragment of *ORC1/CDC6* was amplified by PCR (primers CTOL01 and CTOL02; sequences available on request) and cloned into the vector pZJM (Wang et al., 2000), where it is flanked by opposing T7 promoters and Tet operator sequences. The construct was digested with NotI, allowing integration into the rDNA arrays after transformation. Transformant clones were selected with 10 $\mu g.ml^{-1}$ zeocin (PCF) and 2.5 $\mu g.ml^{-1}$ phleomycin (BSF). To quantify levels of ORC1/CDC6 mRNA, primers CTOL7 and CTOL8 were used; *GPI8* primers (CTOL27 and CTOL28) and *tubulin* primers (CTOL31 and CTOL32) were used as controls. To quantify levels of *BVSG* mRNAs, primer pairs CTOL35/CTOL36, CTOL39/CTOL40, CTOL41/CTOL42, CTOL37/CTOL38, and CTOL33/CTOL345 were used to PCR amplify *VSG13 (VSG427-13)*, *VSG224 (VSG224/427-3)*, *VSG800 (VSG800/427-18)*, *VSGV02 (VSGVO2/427-9)*, and *VSG221 (VSG221/427-2)*, respectively. For *MVSG*s, primer pairs CTOL21/CTOL22, CTOL23/CTOL24, and CTOL25/CTOL26 were used to PCR amplify *VSG*s 1.22, 1.61, and 1.64, respectively. For all qRT-PCRs a master mix for 30 reactions was made in which each reaction had 12.5 μl of SYBR Green PCR Master Mix (Applied Biosystems), 1.0 μl of each primer (300 nM stock), 9.5 μl of dH_2O, and 1.0 μl cDNA (generated by SuperScript First-Strand Synthesis System for RT-PCR [Invitrogen], according to manufacturer's instructions). Reactions were run on an ABI Prism 7000 thermocycler and mRNA levels quantified from amplification according to the manufacturer's instructions; conditions for all reactions were 50°C for 2 min, 95°C for 10 min, followed by 40 cycles of 95°C for 15 s and 60°C for 1 min. Plasmids used for myc tagging of ORC1/CDC6, and the transformation conditions employed, are described elsewhere (Tiengwe et al., 2012).

ChIP and Microarray Hybridization

Microarray design was performed by Roche Diagnostics NimbleGen (USA) using a selected set of *T. brucei* TREU927 DNA sequence files obtained from the Pathogen Sequencing Group at the Wellcome Trust Sanger Institute (Cambridge), chosen to provide coverage of megabase chromosomes and some examples of telomeric sequences. The NimbleGen Tiling ChIP Service (design ID 19441, design name 090109_Tbru_LM_CHIP) was used: 385,816 probes were tiled across all unique regions of the *T. brucei* genome at an average spacing of 61 bp. ChIP was performed from PCF *T. brucei* cells, essentially following Siegel et al. (2009). A total of 10^8 PCF cells were used for each ChIP experiment in 40 ml of SDM79 medium and were crosslinked by incubating them for 20 min at room temperature in 11% formaldehyde (in a buffer containing 50 mM HEPES [pH 7.55], 100 mM NaCl, 1 mM EDTA, and 0.5 mM EGTA). After crosslinking, glycine was added to the mix at a final concentration of 125 mM and centrifuged at 4,000 × *g* for 20 min at 4°C. The cells were then washed with 30 ml ice-cold PBS and centrifuged again at 4,000 × *g* for 20 min at 4°C. After the wash, cells were resuspended in lysis buffer 1 (50 mM HEPES [pH 7.55], 140 mM NaCl, 1.0 mM EDTA, 1.0 mM EGTA, 10% glycerol, 0.25% Triton X-100, 0.5% NP-40, and protease inhibitors), vortexed thoroughly, and rocked on a platform rocker for 10 min. After rocking, the cells were centrifuged at 4,000 × *g* for 20 min at 4°C, and the supernatant was decanted. The cell pellet was next resuspended in lysis buffer 2 (50 mM Tris-HCl [pH 7.8], 200 mM NaCl, 1.0 mM EDTA, 1.0 mM EGTA, and protease inhibitor), and the vortexing and centrifugation were repeated. After decanting the supernatant from this step, the pellet was finally resuspended in 2 ml of lysis buffer 3 (10 mM Tris-HCl [pH 7.8], 100 mM NaCl, 1.0 mM EDTA, 0.1% Na-Deoxycholate, 0.5% N-lauroylsarcosine, and protease inhibitors). Next, the whole-cell lysates were sonicated for 60 cycles (30 s on/30 s off) using a Bioruptor (Diagenode), giving a DNA size range of 0.2–1.0 Kbp (assessed by gel electrophoresis). After sonication, the material was centrifuged at 16,100 × *g* for 10 min at 4°C and a 20 μl aliquot removed to serve as the "input sample." IPs were then performed overnight with 10 μg of anti-Myc monoclonal antibody (Millipore) coupled to M-280 IgG magnetic Dynabeads (Invitrogen). Beads were washed seven times with ice-cold wash buffer (50 mM HEPES [pH 7.55], 500 mM LiCl, 1.0 mM EDTA, 1.0 mM EGTA, 0.7% Na deoxycholate, 1% NP-40, and protease inhibitor), each time collecting the beads and discarding the supernatant, and then washed with 1 ml TE wash buffer (50 mM Tris-HCl [pH 8.0], 50 mM NaCl, and 1.0 mM EDTA). After the TE wash step, the samples were centrifuged for 3 min at 1,000 × *g* at 4°C, and any residual TE wash buffer was carefully removed. A total of 220 μl of elution buffer (50 mM Tris-HCl [pH 8.0], 1% SDS, 10 mM EDTA) was then added to the samples and incubated in a 65°C water bath for 30 min with vortexing every 2–5 min. The beads were then centrifuged for 1 min at 16,100 × *g* at room temperature. A total of 200 μl of the supernatant was removed after the spin step and transferred to a new microcentrifuge tube. This served as the eluate, from which DNA was reverse crosslinked from proteins. To do this, 3 vol of elution buffer was added to the "input sample" and mixed thoroughly. From here the "input sample" was treated like the eluted IP material. Both samples were reverse crosslinked by incubating at 65°C for ~9 hr (not more than 15 hr). After incubation, 8 μl of RNaseA (from a stock of 10 $mg.ml^{-1}$) was added to each sample and mixed by inverting the tubes several times, and this was then incubated at 37°C for 2 hr. Next, 4 μl of Proteinase K (from a stock of 20 $mg.ml^{-1}$) was added to each sample and mixed by inverting the tube several times, and incubated at 55°C for 2 hr. The DNA was then purified using a QIAGEN gel extraction kit and eluted with 60 μl elution buffer, following the manufacturer's protocol. The purified DNA was then repaired using the Quick Blunting kit (NEB) according to manufacturer's protocol. After the repair step, DNA was purified using the QIAquick PCR Purification Kit (QIAGEN), according to manufacturer's instructions, and eluted with 41 μl of H_2O. The purified DNA was then amplified using the Whole Genome Amplification kit (Sigma-Aldrich) and purified using the PCR purification Kit from QIAGEN, both according to manufacturer's instructions. A total of 5 μl of the eluted DNA was run on a 1.5% agarose gel, and the remaining material was quantified using a NanoDrop. For microarray analysis, ~5 μg of input and eluted DNA was used, and labeling, hybridization, and data acquisition were performed by NimbleGen. TbORC1/CDC6-Myc IP DNA was labeled with Cy5 and input sample labeled with Cy3, and these were then cohybridized to the microarray. After cohybridization, data acquisition, and analysis by NimbleGen, the scaled log2 ratio and peak data sets were aligned with the annotated genome sequence coordinates from *T. brucei* strain TREU927 using NimbleGen SignalMap software. For visualization the scaled log2 ratio data were manually adjusted to a minimum value of zero on the y axis of the plots, corresponding to an equal abundance of the Cy5-labeled sample and Cy3-labeled samples; positive log2 ratios correspond to enrichment of Cy5-labeled ChIP DNA relative to the Cy3-labeled input. To analyze the data, the scaled log2 ratio was normalized by searching for four or more probes whose signals were above specified cutoff values (percentage of a hypothetical maximum, which is the mean + 6 [SD]), ranging from 90% to 15%, using a sliding window of 500 bp. The ratio data were then randomized 20 times to evaluate the probability of false positives, allowing the assignment of likely ORC1/CDC6 binding and sites, which were dependent on specified FDRs of ≤0.05 (5% of sites will be incorrect), 0.05–0.1 (5%–10% incorrect), and 0.1–0.2 (10%–20% incorrect).

MFA

PCF *T. brucei* TREU 927 cells were used. To prepare cells for fluorescence-activated cell sorting (FACS), cells were grown in 200 ml of SDM-79 medium to a density of 5 × 10^6 $cells.ml^{-1}$ and harvested by centrifugation at 500 × *g* for 20 min. The cell pellet was resuspended in 0.5 ml of PBS and fixed with 1% formaldehyde in PBS. After quenching the formaldehyde with glycine, cells were permeabilized with 0.01% Triton X-100 in PBS, then incubated for 1 hr at 37°C with 100 $\mu g.ml^{-1}$ RNaseA, and stained with 10 $\mu g.ml^{-1}$ propidium iodide. Cells were sorted by FACS using a Dako Cytomation Mo-Flo high-speed Sorter. A 488 nm laser at 100 mW was used, and emitted light collected through a 630/30 band-pass filter with a photomultiplier tube at 770V. Gating was set on side-scatter, then by the fluorescence measured on a linear scale. Sorted S phase and G2 cell fractions were centrifuged at 1,500 × *g* for 10 min. The cell pellet was resuspended in 300 μl of buffer ATL (QIAGEN) and incubated with 20 μl of Proteinase K solution (QIAGEN) at 65°C overnight to reverse crosslinking, after which DNA was extracted using a QIAamp DNA Micro Kit (QIAGEN) following the manufacturer's instructions. The amount of DNA in each sample was determined using a Qubit 2.0 (Invitrogen), and 200 ng of each was sent for library preparation and Illumina sequencing. Paired-end 100 bp reads were obtained and mapped to the genome of *T. brucei* strain TREU927 (TriTrypDB release 3.3) using Burrows-Wheeler Aligner (BWA) (Li and Durbin, 2010). The read depth for each sample was determined for each nucleotide position along each chromosome, by summing the reads that covered that nucleotide. The read-depth data for each position were then

binned into 2,500 bp sections, and the average value across each 2,500 bp bin was used to calculate the ratio of read-depth between the S and G2 samples, with the baseline for each chromosome normalized to one. For MFA by qPCR, primer pairs were designed to target amplicons of approximately 70 bp situated throughout chromosomes 4, 6, and 8 (primer sequences available on request). Reactions were prepared in quadruplicate using Power SYBR Green PCR Master Mix (Applied Biosystems). Each plate included the same control (an empirically determined region of chromosome 6 that showed no evidence for replication in the G2 sample) used to normalize the results and compare between plates. Reactions were run on an Eppendorf Realplex Mastercycler (in Oxford) and an Applied Biosystems 7500 Real-Time PCR system (in Glasgow), using the same two-step PCR-cycling conditions on each (10 min hold step at 95°C, followed by 40 cycles of 15 s at 95°C, 60 s at 60°C). The fluorescence intensity was recorded at the end of the annealing/extension phase in each cycle. For each amplicon the average Ct value was determined, and the relative amount of DNA present was calculated using the $\Delta\Delta$Ct method by Livak and Schmittgen (2001). Briefly, differences in the total amount of DNA present in each sample are determined by normalizing to a calibrator (the control region on chromosome 6), and the difference between the normalized values for S phase and G2 samples at the region of interest is used to determine the relative quantity of S phase DNA at that position.

RNAseq Analysis of mRNA Abundance

RNA was prepared from PCF Lister 427 cells 96 hr after tetracycline induction of RNAi against ORC1/CDC6 and from control cells that had not been treated with tetracycline. cDNA was then generated from polyA-selected RNA and Illumina Tru-Seq RNA libraries prepared, from which the samples were sequenced on an Illumina GAIIX (performed in the Sir Henry Wellcome Functional Genomics Facility, University of Glasgow). The Illumina reads from each sample were converted to Sanger-encoded quality scores and trimmed such that the median score for each read was >20. The reads were aligned to the genome using Bowtie (Langmead et al., 2009) with parameters that report the best 10 alignments for reads with multiple alignments but remove reads that map to more than 50 locations in the genome (-S -k10−best−strata -m50). This allowed some sensitivity to families of closely related genes but did not allow them to be overrepresented in the further analysis. Gene expression values were calculated as FPKM (fragments per kilobase of exon per million reads mapped) with Cufflinks (Trapnell et al., 2010), using parameters that correct for read bias using the genomic background and reweight reads with multiple alignments (-N -u -b reference.fasta -G reference.gtf). Read depth per position in the genome was generated using the RNA-seq bam files using SAMtools (Li et al., 2009) (SAMtools mpileup -B) and awk. These were loaded into R (http://www.R-project.org/) and normalized using quantile normalization to make the read counts and distributions comparable. To detect those regions where mRNA was expressed and abundance changed, the ratio of read depth for the RNAi-induced to -uninduced samples was then calculated and log2 transformed. To detect those regions where there was no RNAseq reads in one sample, but where reads were detected in the other sample, the positions with zero in either of the samples were set to 0.00001; this highlighted peaks of expression. In each case, reads were binned into 250 bases along the chromosome, and the median depth for each bin was used as the center value. These data were then smoothed, using a running median with a window of 7 to remove some of the background noise, and visualized by SignalMap.

Chromosome Annotation

Megabase chromosomes were demarcated into discrete regions using a custom Perl script. Beginning from one chromosome end, genes along the length of each megabase chromosome were analyzed sequentially, and upon arrival at the coordinates of the first *VSG*, a "*VSG* array" feature was generated. Identification of *RHS*, *VSG* related, and *ESAG*s (but not *ESAG3* because this is also in *VSG* arrays) then generated a "subtelomere-proximal" feature. All hypothetical genes were omitted, and a "core" feature was generated that corresponds with the region encompassing all nonhypothetical genes, and gaps were then joined. Some short regions containing subtelomeric genes present at SSRs in the core of chromosomes were identified, corresponding with where chromosome fusions are believed to have occurred (El-Sayed et al., 2005), and these were annotated as subtelomere features, despite being in the chromosome cores. To demarcate the components of the *VSG* cassette, a distinct Perl script was used. Here, we first searched for 70 bp repeat features, and each repeat was annotated in the genome and manually checked. *VSG* ORF features were then looked for relative to the 70 bp repeats, and this annotation allowed the demarcation of the *VSG* 5′ and 3′ flanks.

ACCESSION NUMBERS

ORC1/CDC6 localization, MFAseq, and RNAseq after ORC1/CDC6 RNAi data are being archived at http://tritrypdb.org.

SUPPLEMENTAL INFORMATION

Supplemental Information includes five figures and one table and can be found with this article online at http://dx.doi.org/10.1016/j.celrep.2012.06.007.

LICENSING INFORMATION

This is an open-access article distributed under the terms of the Creative Commons Attribution 3.0 Unported License (CC-BY; http://creativecommons.org/licenses/by/3.0/legalcode).

ACKNOWLEDGMENTS

This work was supported by the Wellcome Trust (083485). The Wellcome Trust Centre for Molecular Parasitology is supported by core funding from the Wellcome Trust (085349). We thank George Cross and Nicolai Siegel for providing ChIP-seq data for histone H4K10Ac localization, Julie Galbraith and Pawel Herzyk (Glasgow University, Sir Henry Wellcome Functional Genomics facility) for performing the RNAseq analysis, and colleagues in Glasgow and Oxford for discussions.

Received: April 30, 2012
Revised: May 30, 2012
Accepted: June 7, 2012
Published online: July 12, 2012

REFERENCES

Blow, J.J., Ge, X.Q., and Jackson, D.A. (2011). How dormant origins promote complete genome replication. Trends Biochem. Sci. *36*, 405–414.

Bochman, M.L., and Schwacha, A. (2009). The Mcm complex: unwinding the mechanism of a replicative helicase. Microbiol. Mol. Biol. Rev. *73*, 652–683.

Cadoret, J.C., Meisch, F., Hassan-Zadeh, V., Luyten, I., Guillet, C., Duret, L., Quesneville, H., and Prioleau, M.N. (2008). Genome-wide studies highlight indirect links between human replication origins and gene regulation. Proc. Natl. Acad. Sci. USA *105*, 15837–15842.

Cayrou, C., Coulombe, P., Vigneron, A., Stanojcic, S., Ganier, O., Peiffer, I., Rivals, E., Puy, A., Laurent-Chabalier, S., Desprat, R., and Méchali, M. (2011). Genome-scale analysis of metazoan replication origins reveals their organization in specific but flexible sites defined by conserved features. Genome Res. *21*, 1438–1449.

Chuang, R.Y., and Kelly, T.J. (1999). The fission yeast homologue of Orc4p binds to replication origin DNA via multiple AT-hooks. Proc. Natl. Acad. Sci. USA *96*, 2656–2661.

Cliffe, L.J., Siegel, T.N., Marshall, M., Cross, G.A., and Sabatini, R. (2010). Two thymidine hydroxylases differentially regulate the formation of glucosylated DNA at regions flanking polymerase II polycistronic transcription units throughout the genome of *Trypanosoma brucei*. Nucleic Acids Res. *38*, 3923–3935.

Costas, C., de la Paz Sanchez, M., Stroud, H., Yu, Y., Oliveros, J.C., Feng, S., Benguria, A., López-Vidriero, I., Zhang, X., Solano, R., et al. (2011).

Genome-wide mapping of *Arabidopsis thaliana* origins of DNA replication and their associated epigenetic marks. Nat. Struct. Mol. Biol. *18*, 395–400.

Dai, J., Chuang, R.Y., and Kelly, T.J. (2005). DNA replication origins in the *Schizosaccharomyces pombe* genome. Proc. Natl. Acad. Sci. USA *102*, 337–342.

Daniels, J.P., Gull, K., and Wickstead, B. (2010). Cell biology of the trypanosome genome. Microbiol. Mol. Biol. Rev. *74*, 552–569.

Diffley, J.F. (2010). The many faces of redundancy in DNA replication control. Cold Spring Harb. Symp. Quant. Biol. *75*, 135–142.

Diffley, J.F. (2011). Quality control in the initiation of eukaryotic DNA replication. Philos. Trans. R. Soc. Lond. B Biol. Sci. *366*, 3545–3553.

Donti, T.R., Datta, S., Sandoval, P.Y., and Kapler, G.M. (2009). Differential targeting of *Tetrahymena* ORC to ribosomal DNA and non-rDNA replication origins. EMBO J. *28*, 223–233.

Douglas, M.E., and Diffley, J.F. (2012). Replication timing: the early bird catches the worm. Curr. Biol. *22*, R81–R82.

Duncker, B.P., Chesnokov, I.N., and McConkey, B.J. (2009). The origin recognition complex protein family. Genome Biol. *10*, 214.

Eaton, M.L., Galani, K., Kang, S., Bell, S.P., and MacAlpine, D.M. (2010). Conserved nucleosome positioning defines replication origins. Genes Dev. *24*, 748–753.

Ekanayake, D.K., Minning, T., Weatherly, B., Gunasekera, K., Nilsson, D., Tarleton, R., Ochsenreiter, T., and Sabatini, R. (2011). Epigenetic regulation of transcription and virulence in *Trypanosoma cruzi* by O-linked thymine glucosylation of DNA. Mol. Cell. Biol. *31*, 1690–1700.

El-Sayed, N.M., Myler, P.J., Blandin, G., Berriman, M., Crabtree, J., Aggarwal, G., Caler, E., Renauld, H., Worthey, E.A., Hertz-Fowler, C., et al. (2005). Comparative genomics of trypanosomatid parasitic protozoa. Science *309*, 404–409.

Figueiredo, L.M., Cross, G.A., and Janzen, C.J. (2009). Epigenetic regulation in African trypanosomes: a new kid on the block. Nat. Rev. Microbiol. *7*, 504–513.

Gilbert, D.M. (2010). Evaluating genome-scale approaches to eukaryotic DNA replication. Nat. Rev. Genet. *11*, 673–684.

Ginger, M.L., Blundell, P.A., Lewis, A.M., Browitt, A., Günzl, A., and Barry, J.D. (2002). Ex vivo and in vitro identification of a consensus promoter for VSG genes expressed by metacyclic-stage trypanosomes in the tsetse fly. Eukaryot. Cell *1*, 1000–1009.

Godoy, P.D., Nogueira-Junior, L.A., Paes, L.S., Cornejo, A., Martins, R.M., Silber, A.M., Schenkman, S., and Elias, M.C. (2009). Trypanosome prereplication machinery contains a single functional orc1/cdc6 protein, which is typical of archaea. Eukaryot. Cell *8*, 1592–1603.

Haenni, S., Studer, E., Burkard, G.S., and Roditi, I. (2009). Bidirectional silencing of RNA polymerase I transcription by a strand switch region in *Trypanosoma brucei*. Nucleic Acids Res. *37*, 5007–5018.

Hayashi, M., Katou, Y., Itoh, T., Tazumi, A., Yamada, Y., Takahashi, T., Nakagawa, T., Shirahige, K., and Masukata, H. (2007). Genome-wide localization of pre-RC sites and identification of replication origins in fission yeast. EMBO J. *26*, 1327–1339.

Hertz-Fowler, C., Figueiredo, L.M., Quail, M.A., Becker, M., Jackson, A., Bason, N., Brooks, K., Churcher, C., Fahkro, S., Goodhead, I., et al. (2008). Telomeric expression sites are highly conserved in *Trypanosoma brucei*. PLoS One *3*, e3527.

Hickman, M.A., and Rusche, L.N. (2010). Transcriptional silencing functions of the yeast protein Orc1/Sir3 subfunctionalized after gene duplication. Proc. Natl. Acad. Sci. USA *107*, 19384–19389.

Horn, D., and McCulloch, R. (2010). Molecular mechanisms underlying the control of antigenic variation in African trypanosomes. Curr. Opin. Microbiol. *13*, 700–705.

Karnani, N., Taylor, C.M., Malhotra, A., and Dutta, A. (2010). Genomic study of replication initiation in human chromosomes reveals the influence of transcription regulation and chromatin structure on origin selection. Mol. Biol. Cell *21*, 393–404.

Kolev, N.G., Franklin, J.B., Carmi, S., Shi, H., Michaeli, S., and Tschudi, C. (2010). The transcriptome of the human pathogen *Trypanosoma brucei* at single-nucleotide resolution. PLoS Pathog. *6*, e1001090.

Labib, K., and Hodgson, B. (2007). Replication fork barriers: pausing for a break or stalling for time? EMBO Rep. *8*, 346–353.

Langmead, B., Trapnell, C., Pop, M., and Salzberg, S.L. (2009). Ultrafast and memory-efficient alignment of short DNA sequences to the human genome. Genome Biol. *10*, R25.

Leung, K.F., Riley, F.S., Carrington, M., and Field, M.C. (2011). Ubiquitylation and developmental regulation of invariant surface protein expression in trypanosomes. Eukaryot. Cell *10*, 916–931.

Li, H., and Durbin, R. (2010). Fast and accurate long-read alignment with Burrows-Wheeler transform. Bioinformatics *26*, 589–595.

Li, H., Handsaker, B., Wysoker, A., Fennell, T., Ruan, J., Homer, N., Marth, G., Abecasis, G., and Durbin, R.; 1000 Genome Project Data Processing Subgroup. (2009). The Sequence Alignment/Map format and SAMtools. Bioinformatics *25*, 2078–2079.

Livak, K.J., and Schmittgen, T.D. (2001). Analysis of relative gene expression data using real-time quantitative PCR and the 2(-Delta Delta C(T)) Method. Methods *25*, 402–408.

MacAlpine, D.M., Rodríguez, H.K., and Bell, S.P. (2004). Coordination of replication and transcription along a Drosophila chromosome. Genes Dev. *18*, 3094–3105.

MacAlpine, H.K., Gordân, R., Powell, S.K., Hartemink, A.J., and MacAlpine, D.M. (2010). Drosophila ORC localizes to open chromatin and marks sites of cohesin complex loading. Genome Res. *20*, 201–211.

Mancio-Silva, L., Rojas-Meza, A.P., Vargas, M., Scherf, A., and Hernandez-Rivas, R. (2008). Differential association of Orc1 and Sir2 proteins to telomeric domains in *Plasmodium falciparum*. J. Cell Sci. *121*, 2046–2053.

Marcello, L., and Barry, J.D. (2007). From silent genes to noisy populations-dialogue between the genotype and phenotypes of antigenic variation. J. Eukaryot. Microbiol. *54*, 14–17.

Martínez-Calvillo, S., Yan, S., Nguyen, D., Fox, M., Stuart, K., and Myler, P.J. (2003). Transcription of Leishmania major Friedlin chromosome 1 initiates in both directions within a single region. Mol. Cell *11*, 1291–1299.

Martínez-Calvillo, S., Nguyen, D., Stuart, K., and Myler, P.J. (2004). Transcription initiation and termination on Leishmania major chromosome 3. Eukaryot. Cell *3*, 506–517.

Masai, H., Matsumoto, S., You, Z., Yoshizawa-Sugata, N., and Oda, M. (2010). Eukaryotic chromosome DNA replication: where, when, and how? Annu. Rev. Biochem. *79*, 89–130.

Méchali, M. (2010). Eukaryotic DNA replication origins: many choices for appropriate answers. Nat. Rev. Mol. Cell Biol. *11*, 728–738.

Merrikh, H., Machón, C., Grainger, W.H., Grossman, A.D., and Soultanas, P. (2011). Co-directional replication-transcription conflicts lead to replication restart. Nature *470*, 554–557.

Obado, S.O., Bot, C., Nilsson, D., Andersson, B., and Kelly, J.M. (2007). Repetitive DNA is associated with centromeric domains in *Trypanosoma brucei* but not *Trypanosoma cruzi*. Genome Biol. *8*, R37.

Prasanth, S.G., Shen, Z., Prasanth, K.V., and Stillman, B. (2010). Human origin recognition complex is essential for HP1 binding to chromatin and heterochromatin organization. Proc. Natl. Acad. Sci. USA *107*, 15093–15098.

Respuela, P., Ferella, M., Rada-Iglesias, A., and Aslund, L. (2008). Histone acetylation and methylation at sites initiating divergent polycistronic transcription in *Trypanosoma cruzi*. J. Biol. Chem. *283*, 15884–15892.

Robinson, N.P., and Bell, S.D. (2005). Origins of DNA replication in the three domains of life. FEBS J. *272*, 3757–3766.

Sasaki, T., and Gilbert, D.M. (2007). The many faces of the origin recognition complex. Curr. Opin. Cell Biol. *19*, 337–343.

Sequeira-Mendes, J., Díaz-Uriarte, R., Apedaile, A., Huntley, D., Brockdorff, N., and Gómez, M. (2009). Transcription initiation activity sets replication origin efficiency in mammalian cells. PLoS Genet. *5*, e1000446.

Siegel, T.N., Hekstra, D.R., Kemp, L.E., Figueiredo, L.M., Lowell, J.E., Fenyo, D., Wang, X., Dewell, S., and Cross, G.A. (2009). Four histone variants mark the boundaries of polycistronic transcription units in *Trypanosoma brucei*. Genes Dev. *23*, 1063–1076.

Siegel, T.N., Hekstra, D.R., Wang, X., Dewell, S., and Cross, G.A. (2010). Genome-wide analysis of mRNA abundance in two life-cycle stages of *Trypanosoma brucei* and identification of splicing and polyadenylation sites. Nucleic Acids Res. *38*, 4946–4957.

Tardat, M., Brustel, J., Kirsh, O., Lefevbre, C., Callanan, M., Sardet, C., and Julien, E. (2010). The histone H4 Lys 20 methyltransferase PR-Set7 regulates replication origins in mammalian cells. Nat. Cell Biol. *12*, 1086–1093.

Thomas, S., Green, A., Sturm, N.R., Campbell, D.A., and Myler, P.J. (2009). Histone acetylations mark origins of polycistronic transcription in Leishmania major. BMC Genomics *10*, 152.

Tiengwe, C., Marcello, L., Farr, H., Gadelha, C., Burchmore, R., Barry, J.D., Bell, S.D., and McCulloch, R. (2012). Identification of ORC1/CDC6-interacting factors in *Trypanosoma brucei* reveals critical features of origin recognition complex architecture. PLoS One *7*, e32674.

Trapnell, C., Williams, B.A., Pertea, G., Mortazavi, A., Kwan, G., van Baren, M.J., Salzberg, S.L., Wold, B.J., and Pachter, L. (2010). Transcript assembly and quantification by RNA-Seq reveals unannotated transcripts and isoform switching during cell differentiation. Nat. Biotechnol. *28*, 511–515.

Wang, Z., Morris, J.C., Drew, M.E., and Englund, P.T. (2000). Inhibition of *Trypanosoma brucei* gene expression by RNA interference using an integratable vector with opposing T7 promoters. J. Biol. Chem. *275*, 40174–40179.

Wickstead, B., Ersfeld, K., and Gull, K. (2004). The small chromosomes of *Trypanosoma brucei* involved in antigenic variation are constructed around repetitive palindromes. Genome Res. *14*, 1014–1024.

Wirtz, E., Leal, S., Ochatt, C., and Cross, G.A. (1999). A tightly regulated inducible expression system for conditional gene knock-outs and dominant-negative genetics in *Trypanosoma brucei*. Mol. Biochem. Parasitol. *99*, 89–101.

Wright, J.R., Siegel, T.N., and Cross, G.A. (2010). Histone H3 trimethylated at lysine 4 is enriched at probable transcription start sites in *Trypanosoma brucei*. Mol. Biochem. Parasitol. *172*, 141–144.

Wyrick, J.J., Aparicio, J.G., Chen, T., Barnett, J.D., Jennings, E.G., Young, R.A., Bell, S.P., and Aparicio, O.M. (2001). Genome-wide distribution of ORC and MCM proteins in *S. cerevisiae*: high-resolution mapping of replication origins. Science *294*, 2357–2360.

Yabuki, N., Terashima, H., and Kitada, K. (2002). Mapping of early firing origins on a replication profile of budding yeast. Genes Cells *7*, 781–789.

Supplemental Information

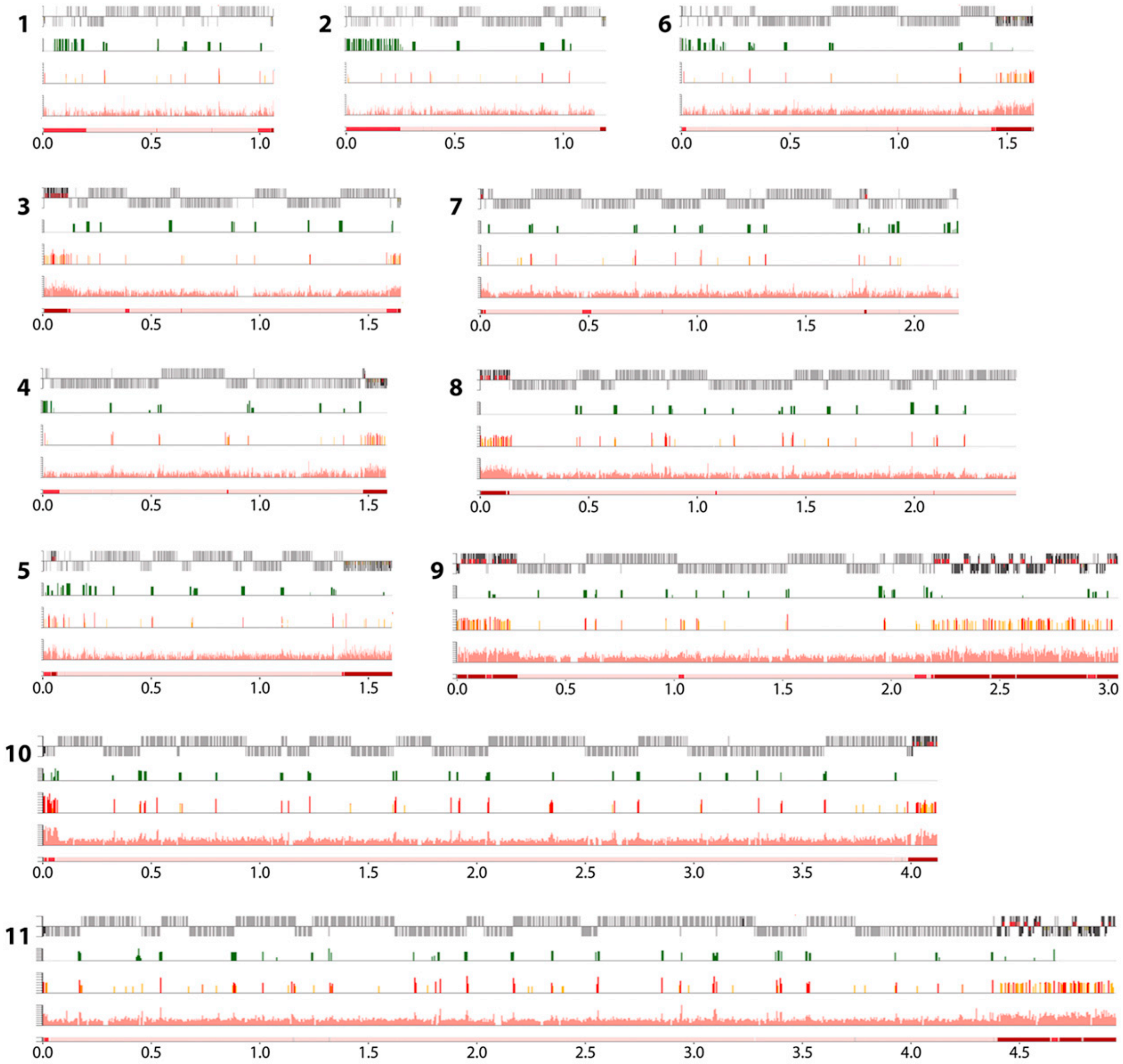

Figure S1. Mapping ORC1/CDC6 Binding Sites in the *T. brucei* Megabase Chromosomes, Related to Figures 1 and 2

TbORC1/CDC6-Myc ChIP and input DNA were labeled with Cy5 and Cy3, respectively, and co-hybridized to a *T. brucei* 385K tiled array. Megabase chromosomes are shown to scale (numbers denote chromosome sizes in Mbp), and each represented in the same way. The bottom panel delineates the chromosome into 'core', 'subtelomere-proximal' and 'VSG array' sequences (light pink, pink and red, respectively). The location of CDSs (gray boxes) along the chromosome is shown in the top panel; genes above the line are transcribed toward the right, and those below are transcribed toward the left. Enrichment of ORC1/CDC6-bound DNA relative to the input is shown in the second bottom panel; values are plotted as the log2-ratio of sample/input (maximum *y* axis scale value 2), and were calculated over a 500 bp sliding window. In the panel above, predicted ORC1/CDC6 binding sites are identified as 'peaks', which are shown by vertical lines colored to indicate the likelihood of being an ORC1/CDC6 binding site based on three categories of False Discovery Rate (FDR): red indicates the highest confidence (FDR $\leq$ 0.05), and orange and yellow decreasing confidence (FDRs of 0.05-0.1 and 0.1-0.2, respectively). In the second top panel, sites of H4K10Ac localization are indicated as green vertical lines; these data are also shown as a log2-ratio (maximum *y* axis scale value 150) and are derived from ChIP-seq data of Siegel et al. (2009), identifying positions of likely transcription start sites, with the width of the lines indicating the areas of the chromosome covered by the modified histone.

Cell PRESS Open ACCESS

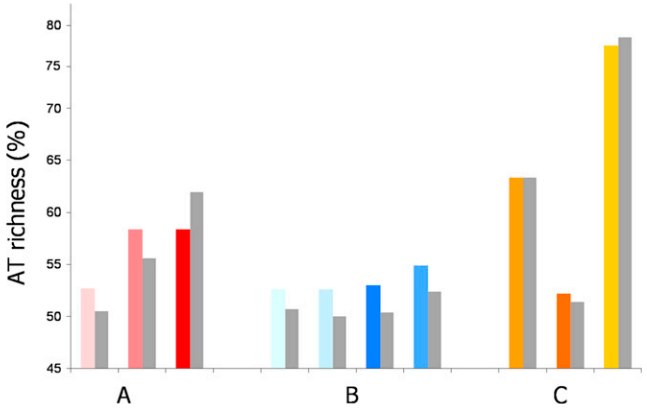

Figure S2. AT Content of ORC1/CDC6 Binding Sites, Related to Figure 3

The graph depicts the percent of *T. brucei* genome sequence composed of AT residues, comparing predicted ORC1/CDC6 binding sites (colored bars follow the scheme in Figure 2; see below) to unbound sequence (gray bars) in specific components of the genome. In the whole genome (A) AT richness is analyzed for core (light pink), subtelomere-proximal (pink) and *VSG* array (red) sequence components. Within the core (B), AT richness is compared in strand switch regions (divergent, dark blue; convergent, mid-blue) and in transcribed regions (forward strand, light blue; reverse strand, very light blue). Finally, AT richness is examined for specific *VSG* array components (C): 5′ *VSG* flank (light orange), *VSG* CDS (dark orange), and 3′ *VSG* flank (yellow).

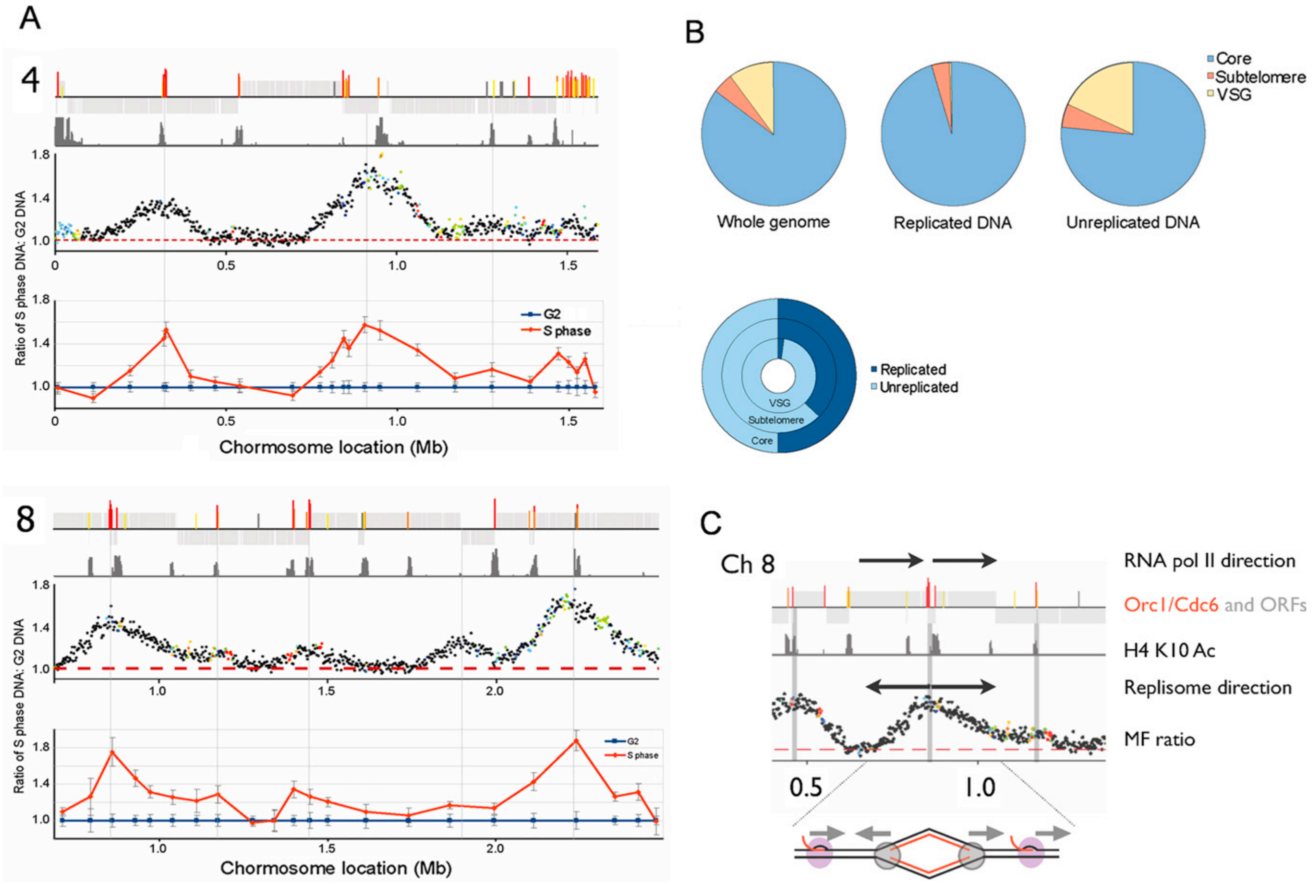

Figure S3. *T. brucei* Origins of Replication, Related to Figure 4

(A) *T. brucei* replication origins were mapped by Marker Frequency Analysis (MFA) using quantitative PCR; MFA results are shown for chromosome 4 (upper diagram) and for ~3 Mbp of chromosome 8 (lower diagram). For each chromosome the data is depicted in the same way. The upper three panels are derived from Figure 3, and show, respectively, predicted ORC1/CDC6 binding sites (red, orange and yellow lines) within the CDS (gray boxes), localization of H4K10Ac (green lines; derived from ChIP-seq data of Siegel et al. (2009), and the ratio of sequencing read-depth between the S phase and G2 DNA samples (each dot represents 2500 bp). The lowest panel shows qPCR at a number of loci along the chromosomes. At each locus the relative quantity of S phase (orange) and G2 phase (blue) DNA is shown, derived from comparison with a control region on chromosome 6 (not shown); G2 values at each locus are set at 1, and the S phase samples shown as a proportion of that value (vertical lines indicate standard deviation from four experimental repeats).

(B) Replication timing of core and subtelomeric elements of the *T. brucei* genome. Pie charts in the upper panel depict the relative proportion of DNA sequences that are found in the core (blue), the subtelomere-proximal (orange) and *VSG* array components (yellow) of the nuclear genome. The whole genome is analyzed in the leftmost chart, and compared with the proportions of the three components in replicated (middle) and unreplicated (right) DNA (data derived from the marker frequency analysis data in Figure 3). In the lower panel the percentage of replicated (dark blue) and unreplicated (light blue) DNA in the three components of the genome is compared as concentric circles (a complete circle represents 100%).

(C) Conflict between DNA replication and transcription in the *T. brucei* genome. Diagram shows Marker Frequency Analysis (MFA) data focused on an origin in chromosome 8, which initiates from an ORC1/CDC6 binding site at a region between two DGCs that are transcribed in the same direction (RNA pol II direction; arrows). Replication direction is indicated, and the annotation of the ORC1/CDC6 binding sites, ORFS, histone H4K10Ac localization and MFAseq data are as described in Figure 3. The cartoon below shows the clash between transcription and the replication fork to the left of the origin, and co-linearity of the processes to the right.

Cell PRESS
Open ACCESS

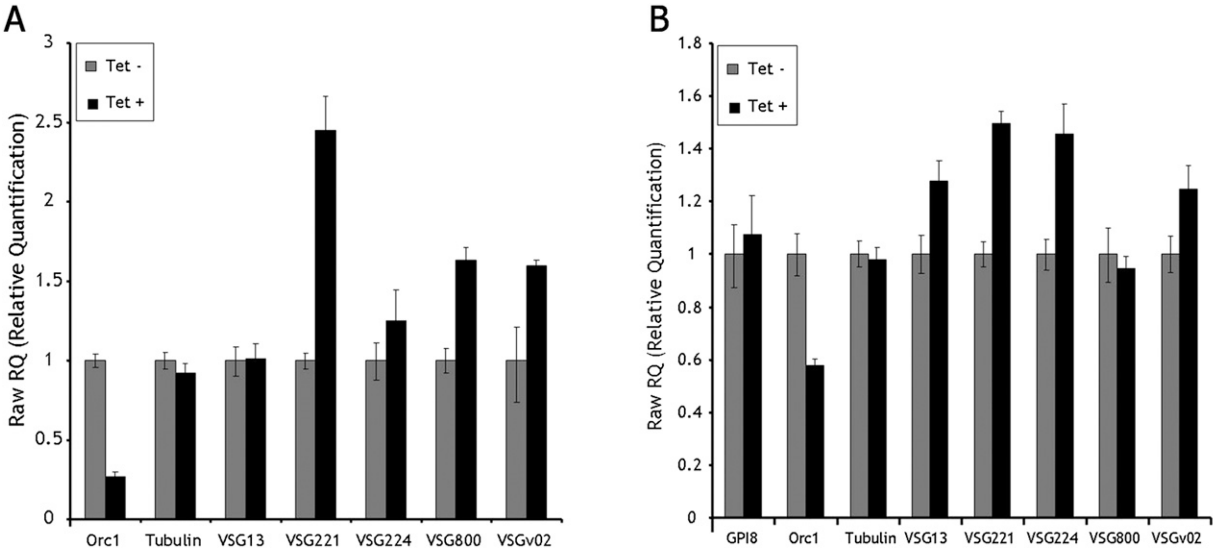

Figure S4. Expression Levels of Bloodstream *VSG* Genes after ORC1/CDC6 RNAi, Related to Figure 5

mRNA abundance was measured in procyclic form cells (A) or bloodstream form cells (B) by quantitative RT-PCR, comparing mRNA levels for five *VSG* genes found in bloodstream form *VSG* expression sites: *VSG13 (VSG427-13), VSG224 (VSG 427-3), (VSG 427-18), VSGV02 (VSG 427-9)* and *VSG221 (427-2)* (Hertz-Fowler et al., 2008). For each *VSG*, RT-PCR was measured relative to *GPI8* as an endogenous control, before (Tet-, gray bar) and after RNAi induction (Tet+, black bar) by addition of tetracycline (Tet). In each case the level of mRNA of the uninduced sample is shown as 1 and the mRNA level in the RNAi-induced sample shown relative to that; vertical lines indicate standard deviation from three experiments. *ORC1/CDC6* mRNA levels are also shown (indicated by Orc1), and tubulin mRNA levels are also shown as a further control.

Cell
PRESS
Open
ACCESS

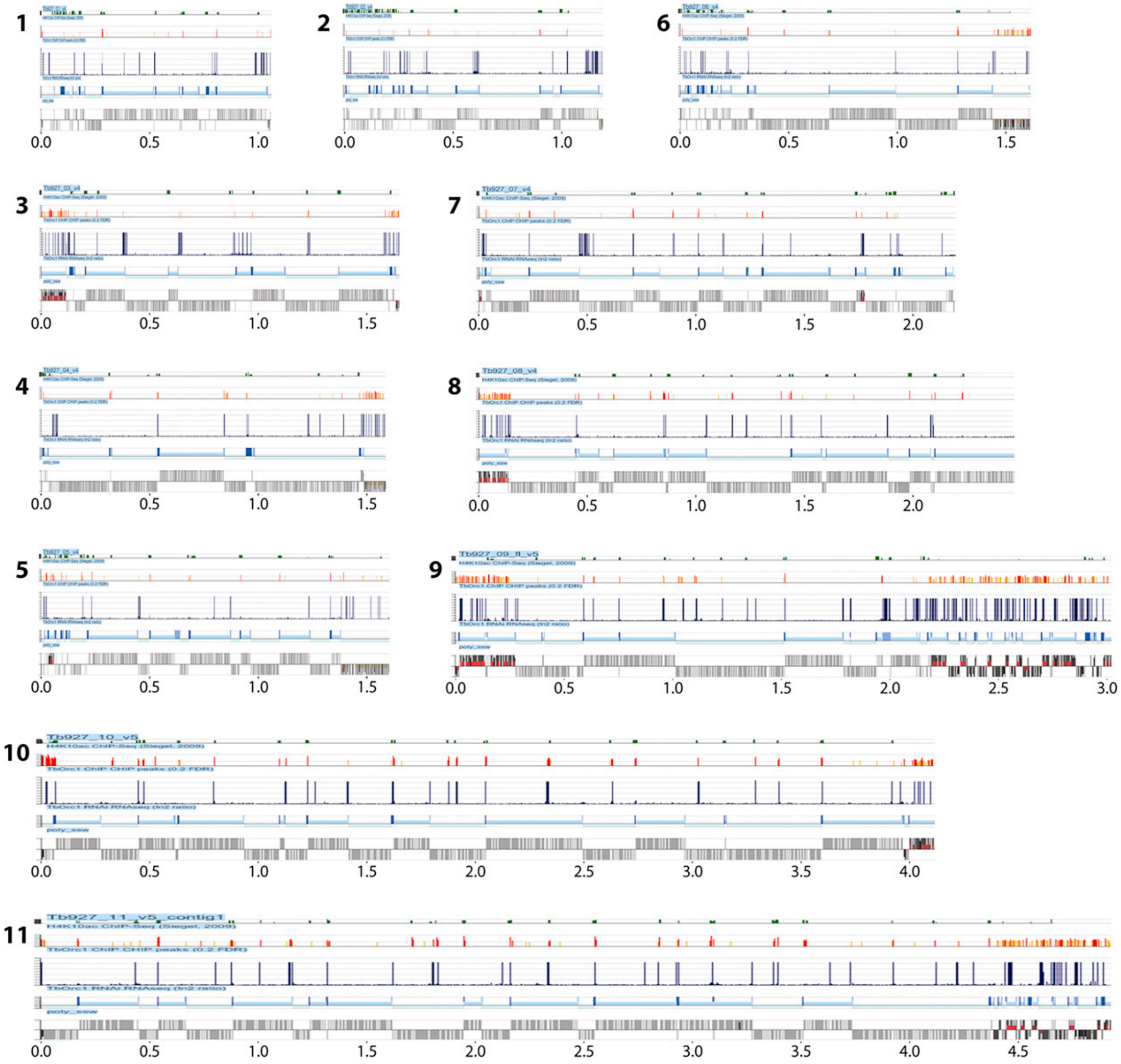

Figure S5. Effect of ORC1/CDC6 RNAi on mRNA Abundance throughout the *T. brucei* Megabase Chromosomes, Related to Figure 6

The effect of ORC1/CDC6 RNAi on mRNA abundance is shown for all chromosomes (1-11, shown to scale; numbers below each chromosome indicate size in Mbp). Each chromosome diagram follows the same scheme. *Top panel*: H4K10Ac binding sites are shown as peaks (green lines); the *y* axis shows max log2 ratio in 250 bp window. *Second panel*: predicted locations of ORC1/CDC6 binding sites (red, orange or yellow lines, as in Figure 1). *Middle panel*: shows chromosome positions where RNA sequence tags were detectable in the RNAi induced sample but were absent in the uninduced sample; locations of RNAi-induced RNA sequence tags are indicated by vertical lines (height is arbitrary, and width is shown in 250 bp windows). *Fourth panel*: details the direction of transcription of the genes (light blue indicates a directional gene cluster transcribed toward the right, and very light blue a cluster transcribed toward the left), and highlights the positions of convergent (mid blue box) and divergent (dark blue box) strand switch regions (SSRs). *Bottom panel*: the locations of CDS (gray boxes) are shown.
